# Dexmedetomidine as a Sedative Agent in Critically Ill Patients: A Meta-Analysis of Randomized Controlled Trials

**DOI:** 10.1371/journal.pone.0082913

**Published:** 2013-12-31

**Authors:** Laura Pasin, Teresa Greco, Paolo Feltracco, Annalisa Vittorio, Caetano Nigro Neto, Luca Cabrini, Giovanni Landoni, Gabriele Finco, Alberto Zangrillo

**Affiliations:** 1 Anesthesia and Intensive Care Department, San Raffaele Scientific Institute, Milan, Italy; 2 Department of Pharmacology and Anesthesiology, University Hospital of Padua, Padua, Italy; 3 Outcomes Research Consortium, Cleveland, Ohio, United States of America; 4 Department of Medical Sciences “M. Aresu”, Cagliari University, Cagliari, Italy; University of Colorado, United States of America

## Abstract

**Introduction:**

The effect of dexmedetomidine on length of intensive care unit (ICU) stay and time to extubation is still unclear.

**Materials and Methods:**

Pertinent studies were independently searched in BioMedCentral, PubMed, Embase, and the Cochrane Central Register of clinical trials (updated February first 2013). Randomized studies (dexmedetomidine versus any comparator) were included if including patients mechanically ventilated in an intensive care unit (ICU). Co-primary endpoints were the length of ICU stay (days) and time to extubation (hours). Secondary endpoint was mortality rate at the longest follow-up available.

**Results:**

The 27 included manuscripts (28 trials) randomized 3,648 patients (1,870 to dexmedetomidine and 1,778 to control). Overall analysis showed that the use of dexmedetomidine was associated with a significant reduction in length of ICU stay (weighted mean difference (WMD) = −0.79 [−1.17 to −0.40] days, p for effect <0.001) and of time to extubation (WMD = −2.74 [−3.80 to −1.65] hours, p for effect <0.001). Mortality was not different between dexmedetomidine and controls (risk ratio = 1.00 [0.84 to 1.21], p for effect = 0.9). High heterogeneity between included studies was found.

**Conclusions:**

This meta-analysis of randomized controlled studies suggests that dexmedetomidine could help to reduce ICU stay and time to extubation, in critically ill patients even if high heterogeneity between studies might confound the interpretation of these results.

## Introduction

Dexmedetomidine was approved by the Food and Drug Administration (FDA) at the end of 1999 as a short-term medication (<24 hours) for analgesia and sedation in mechanical ventilated intensive care unit (ICU) patients. In 2008, the FDA approved a new indication in non intubated patients requiring sedation before and/or during surgical and non-surgical procedures. Dexmedetomidine is a highly selective a2-adrenergic receptor agonist, which binds to transmembrane G protein-binding adrenoreceptors in the periphery (α2A), brain and spinal cord (α2B, α2C) tissues [1]. In contrast to other sedative agents, dexmedetomidine, by acting on a2 receptors in the locus caeruleus [2], has potential analgesic effects [3] without respiratory depression [4], [5]. Only one meta-analysis of randomized controlled trials (RCTs) [6] was published so far: Tan and Ho reported a reduction in length of ICU stay, but not in duration of time to extubation when dexmedetomidine was compared with alternative sedative agents.

Since several RCTs [7]–[14], including two large ones [8], were recently published, and one further RCT [15] was not included in the previous meta-analysis [6] we decided to perform an updated meta-analysis of all the RCTs ever performed on dexmedetomidine versus any comparator in the ICU setting to evaluate time to extubation, ICU stay and survival.

## Materials and Methods

### Search Strategy

Pertinent studies were independently searched in BioMedCentral, PubMed, Embase, and the Cochrane Central Register of clinical trials (updated February 1^st^ 2013) by four trained investigators. The full PubMed search strategy aimed to include any RCTs ever performed in humans with dexmedetomidine in any clinical setting and is presented in the supplemental material (Text S1). In addition, we employed backward snowballing (i.e., scanning of references of retrieved articles and pertinent reviews) and contacted international experts for further studies with no language restriction.

### Study Selection

References were first independently examined at a title/abstract level by four investigators, with divergences resolved by consensus, and then, if potentially pertinent, retrieved as complete articles. The following inclusion criteria were used for potentially relevant studies: random allocation to treatment (dexmedetomidine versus any comparator with no restrictions on dose or time of administration); studies involving patients who required mechanical ventilation in an ICU. The exclusion criteria were duplicate publications (in this case we referred to the first article published while retrieved data from the article with the longest follow-up available), non-adult patients and lack of data on all of the following: ICU stay, time to extubation and mortality. Two investigators independently assessed compliance to selection criteria and selected studies for the final analysis, with divergences resolved by consensus.

### Data Abstraction and Study

Baseline, procedural, and outcome data were independently abstracted by four trained investigators (table 1 and table 2). If a trial reported multiple comparisons [25], [34], the comparators were aggregated as a single control group. At least two separate attempts at contacting original authors were made in cases of missing data. The co-primary endpoints of the present review were the length of ICU stay (days) and time to extubation (hours from randomization to extubation).

**Table 1:** Description of the 28 trials included in the meta-analysis.

First author	Year	Setting	Dex patients	Control patients	Comparator	Comparator dose	Follow-up
Aziz AN [7]	2011	Cardiac surgery	14	14	Morphine	4.6–46 µg/kg/h	24 hours
Corbett SM [21]	2005	Cardiac surgery	43	46	Propofol	0.2–0.7 µg/kg/h or 5–75 µg/kg/min	ICU stay
Elbaradie S [22]	2004	Major surgeries	30	30	Propofol	Bolus dose of 1 mg/kg followed by an infusion of 0.5–1 mg/kg/h	24 hours after commencement of sedative infusions
Esmaoglu A [23]	2009	Post caesarean eclampsia	20	20	Midazolam	Loading dose of 0.05 mg/kg followed by an infusion of 0.1 mg/kg/h	ICU stay
Herr DL [24]	2003	Cardiac surgery	148	147	Propofol	NA	24 hours after discharge from ICU
Jakob SM MIDEX [8]	2012	ICU	249	251	Midazolam	0.03–0.2 mg/kg/h	45 days
Jakob SM PRODEX [8]	2012	ICU	251	247	Propofol	0.3–4.0 mg/kg/h	45 days
Khalil MA [14]	2012	Cardiac surgery	25	25	Placebo	Loading dose 1 µg/kg over 10 minutes followed by a maintenance infusion of 0.5 µg/kg/h	Hospital stay
Leino K [9]	2011	Cardiac surgery	44	43	Placebo	39 ml/h for 20 min, 24.5 ml/h for 40 minutes, 14 ml/h for 60 min, 10.5 ml/h for 120 min and then 7 ml/h	48 hours after catheter insertion
Maldonado JR [25]	2009	Cardiac surgery	4040	3840	Propofol, midazolam	Propofol: 25–50 µg/kg/min; Midazolam: 0.5–2 mg/h	Hospital stay
Martin E [26]	2003	ICU	203	198	Placebo	1 µg/kg for 10 min (loading dose) and then 0.4 µg/kg/h. The latter rate could be adjusted within the range of 0.2 to 0.7 µg/kg/h	24 hours from infusion end
Memis D [27]	2006	ICU	12	12	Propofol	2 mg/kg/h over 5-h infusion	ICU stay
Memis D [28]	2007	ICU	20	20	Midazolam	Loading dose of 0.2 mg/kg over 10 min followed by 0.1–0.5 mg/kg/h infusion	ICU stay
Memis D [29]	2009	ICU	20	20	Propofol	1 mg/kg over 15 min followed by a maintenance dose of 1 to 3 mg/kg per hour	ICU stay
MendaF[10]	2010	Cardiac surgery	15	15	Placebo	1 µg/kg in 15 min	ICU stay
Ozkan N [30]	2007	Cardiac surgery	20	20	Midazolam	0.05–0.07 mg/kg/h	24 hours post extubation
Pandharipande PP [31]	2007	ICU	52	51	Lorazepam	Maximum 10 mg/hr	12 months
Reade MC [32]	2009	ICU	10	10	Haloperidol	0.5–2 mg/hour preceded by a loading dose of 2.5 mg if desired	Hospital stay
Riker RR [33]	2009	ICU	244	122	Midazolam	Loading dose 0.05 mg/kg then infusion rate 0.02–0.1 mg/kg/h	30 days
Ruokonen E [34]	2009	ICU	41	44	Propofol Midazolam	Propofol: 2.4 mg/kg/h for 1 h and then adjusted stepwise at 0.8, 1.6, 2.4, 3.2, and 4.0 mg/kg/h; Midazolam: boluses (1–2 mg), starting at 3 boluses per hour for 1 h, and thereafter 1–4 boluses per h, and if not sufficient as continuous infusion	45 days
Sahin N [15]	2005	Cardiac surgery	15	15	Midazolam	0.1 mg/kg/h intraoperative; 0.5–1 µg/kg/min ICU	12 hours postoperatory
Shehabi Y [35]	2009	Cardiac surgery	154	152	Morphine	10–70 µg/kg/ml	Hospital stay
Tasdogan M [36]	2009	Abdominal surgery	20	20	Propofol	1 mg/kg over 15 minutes followed by a maintenance dose of 1–3 mg/kg/h	25 days
Terao Y [11]	2012	Cervical spine surgery	16	16	Propofol	0.1 mg/kg/min for 10 minutes as a loading dose, followed by a continuous infusion at 1 mg/kg/h	Hospital stay
Triltsch AE [37]	2002	ICU	15	15	Placebo	Loading dose of 6 µg/kg/h 1 for 10 min; maintenance infusion of 0.1–0.7 µg/kg/h	24 hours after the end of study drug infusion
Venn RM [38]	2001	Major surgery	10	10	Propofol	1–3 mg/kg/h after loading dose of up to 1 mg/kg over 10 min	35 days
Wan LJ [12]	2011	ICU	102	98	Midazolam	NA	24 hours
Yao L [13]	2010	ICU	35	38	Midazolam	Loading dose (0.06 mg/kg) and then maintained with 0.04–0.20 mg/kg/h	Time on mechanical ventilation

Dex: dexmedetomidine; ICU: Intensive Care Unit; NA: not available; RASS: Richmond Agitation Sedation Scale; BIS: BispectralIndex ; MAAS: Motor Activity Assessment Scale.

**Table 2:** Doses, sedation scales and target sedation levels.

First author	Study endpoint	Dexmedetomidine dose	Start study drug	Stop study drug
Aziz NA [7]	Sedation quality	0.03–0.25 µg/kg/h	ICU arrival	After 24 hours
Corbett SM [17]	Sedation quality	Loading dose of 1 µg/kg in 15 min, followed by a 0.4/µg/kg/h infusion	During surgery, after CPB	Propofol was discontinued before extubation while dexmedetomidine was continued for up to 1 hour after extubation
Elbaradie S [22]	Sedation quality	Loading dose of 2.5 µg/kgin10 min followed by a 0.2–0.5 µg/kg/h infusion	ICU arrival	Before extubation
Esmaoglu A [23]	Sedation quality	Loading dose of 1 µg/kg in 20 min followed by a0.7 µg/kg/h infusion	ICU arrival	NA
Herr DL [24]	Sedation quality	Loading dose of 1 µg/kg in20 min followed by a0.4 µg/kg/h infusion. After transfer to the ICU, the infusion rate was titrated in the range of 0.2 to 0.7 µg/kg/h	Sternal closure	6–24 hours after extubation
Jakob SM MIDEX [8]	Sedation quality	0.2–1.4 µg/kg/h	Within 72 hours after ICU admission	Extubation, 14 days maximum
Jakob SM PRODEX [8]	Sedation quality	0.2–1.4 µg/kg/h	Within 72 hours after ICU admission	Extubation, 14 days maximum
Khalil MA [14]	Sedation quality	Loading dose of 1 µg/kg in 10 minutes followed by a 0.5 µg/kg/h infusion	After induction of general anaesthesia	After stabilization of haemodynamics in the ICU
Leino K [9]	Renal effects	Five-step infusion of 4 µg/ml with the following decreasing infusion rate: 39 ml/h for 20 min, 24.5 ml/h for 40 min, 14 ml/h for 60 min, 10.5 ml/h for 120 min and then 7 ml/h (rates needed to achieve a pseudo steady-state plasma concentration of 0.60 µg/ml)	Immediately after anaesthesia induction	4 h after ICU arrival
Maldonado JR [25]	Sedation quality	Loading dose of 0,4 µg/kg followed by 0.2–0.7 µg/kg/h	After CPB weaning	Maximum 24 h
Martin E [26]	Sedation quality	Loading dose of 1 µg/kg in 10 min followed by 0.4 µg/kg/h. The latter rate could be adjusted within the range of 0.2 to 0.7 µg/kg/h	Within 1 hour after ICU admission	For a minimum of 6 hours post extubation; total time was <24 hours
Memis D [27]	Gastric emptying	Loading dose of 2.5 µg/kg in 10 min followed by 0.2 µg kg/h over 5 h infusion	Within 4 hours after ICU admission	5 hours
Memis D [28]	Inflammatory responses and gastric intramucosal pH	Loading dose of 1 µg/kg in 10 min followed by 0.2–2.5 µg/kg over 24 h infusion	ICU	NA
Memis D [29]	Indocyanine green elimination	Loading dose of 1 µg/kg in 10 min followed by a maintenance of 0.2–2.5 µg/kg/h	NA	24 hours
Menda F [10]	Haemodynamic response to endotracheal intubation	1 µg/kg in 15 min	Anaesthesia induction	NA
Ozkan N [30]	Haemodynamics and mixed venous oxygen saturation	Loading dose of 1 µg/kg followed by 0.2–0.4 µg/kg/h	Anaesthesia induction	NA
Pandharipande PP [31]	Sedation quality	Maximum 1.5 µg/kg/hr	ICU	Until extubation, for maximum 120 hours
Reade MC [32]	Sedation quality	Loading dose of 1.0 µg/kg in 20 min (if desired) followed by 0.2–0.7 µg/kg/hour	ICU	As long as clinically indicated, including following extubation if required
Riker RR [33]	Sedation quality	Loading dose of 1 µg/kg followed by 0.2–1.4 µg/kg/h	Within 96 hours after intubation	Extubation, 30 days maximum
Ruokonen E [34]	Sedation quality	0.8 µg/kg/h for 1 h and then adjusted stepwise at 0.25, 0.5, 0.8, 1.1, and 1.4 µg/kg/h	Within 72 hours after ICU admission	Maximum 14 days
Sahin N [15]	Sedation quality and haemodynamics	0.4 µg/kg/h intraoperative; 0.2–0.4 µg/kg/h in ICU	Anesthesia induction	45 hours after extubation
Shehabi Y [35]	Sedation quality	0.1–0.7 µg/kg/ml	Within 1 hour after ICU admission	Removal of chest drains, maximum 48 hours
Tasdogan M [36]	Inflammatory responses and intra abdominal pressure	Loading dose of 1 µg/kg in 10 min followed by 0.2–2.5 µg/kg/h	ICU arrival	24 hours
Terao Y [11]	Sedation quality	Loading dose of 0.1 µg/kg/min in 10 minutes followed by 0.4 µg/kg/h	ICU arrival	First postoperative morning
Triltsch AE [37]	Sedation quality	Loading dose of 6 µg/kg/h in 10 min followed by 0.1–0.7 µg/kg/h	Within 1 hour after ICU admission	6–7 hours after extubation, maximum overall 72 h
Venn RM [38]	Sedation quality	Loading dose of 2,5 µg/kg/h followed by 0,2–2,5 µg/kg/h	ICU arrival	Extubation
Wan LJ [12]	Sedation quality	NA	NA	NA
Yao L [13]	Sedation quality	Loading dose of 1 µg/kg in 10 min followed by 0.2–0.7 µg/kg/h	NA	NA

ICU: Intensive Care Unit; CPB: cardiopulmonary bypass; NA: not available.

The secondary endpoint was mortality rate at the longest follow-up available. Adverse effects (hypotension and bradycardia as per author definition) were also analysed. Further endpoints included the number of patients requiring rescue doses of analgesic (opioids) or sedative (propofol, benzodiazepines, or any antipsychotics) drugs and the number of patients completely comfortable during ICU stay.

The internal validity and risk of bias of included trials was appraised by two independent reviewers according to the latest version of the “Risk of bias assessment tool” developed by The Cochrane collaboration [16], with divergences resolved by consensus. Publication bias was assessed by visually inspecting funnel plots and scatter plots and by analytical appraisal based on the Egger's linear regression test and on the Peters' test for asymmetry. According to the Egger [17] or Peters [18] methods for publication bias evaluation, a two-sided p value of 0.10 or less was regarded as significant.

### Data Analysis and Synthesis

Computations were performed with Stata release 11, College Station, TX) and SAS 2002–08 program (release 9.2, SAS Institute, Inc, Cary, NC). Hypothesis of statistical heterogeneity was tested by means of Cochran Q test, with statistical significance set at the two-tailed 0.10 level, whereas extent of statistical consistency was measured with I^2^, defined as 100%×(Q-df)/Q, where Q is Cochran's heterogeneity statistic and df the degrees of freedom. Binary outcomes from individual studies were analysed to compute individual and pooled risk ratio (RR) with pertinent 95% confidence interval (CI), by means of inverse variance method and with a fixed-effect model in case of low statistical inconsistency (I^2^<25%) or with random-effect model (which better accommodates clinical and statistical variations) in case of moderate or high statistical inconsistency (I^2^>25%). Standardized mean differences (SMD), or weighted mean difference (WMD), and 95% confidence intervals were computed for continuous variables using the same models as just described. To evaluate if the small study effect had an influence on the treatment effect estimate, in case of evidence of between-study heterogeneity (I^2^>25), we compared the results of both fixed and random effect models. Sensitivity analyses were performed by sequentially removing each study and reanalysing the remaining dataset (producing a new analysis for each study removed) and by analysing only data from blinded studies and studies with low risk of bias.

Statistical significance was set at the two-tailed 0.05 level for hypothesis testing. Unadjusted p values are reported throughout. This study was performed in compliance with The Cochrane Collaboration and Preferred Reporting Items for Systematic Reviews and Meta-Analyses guidelines [16], [19], [20] (Checklist S1).

## Results

### Study Characteristics

**Figure 1:**
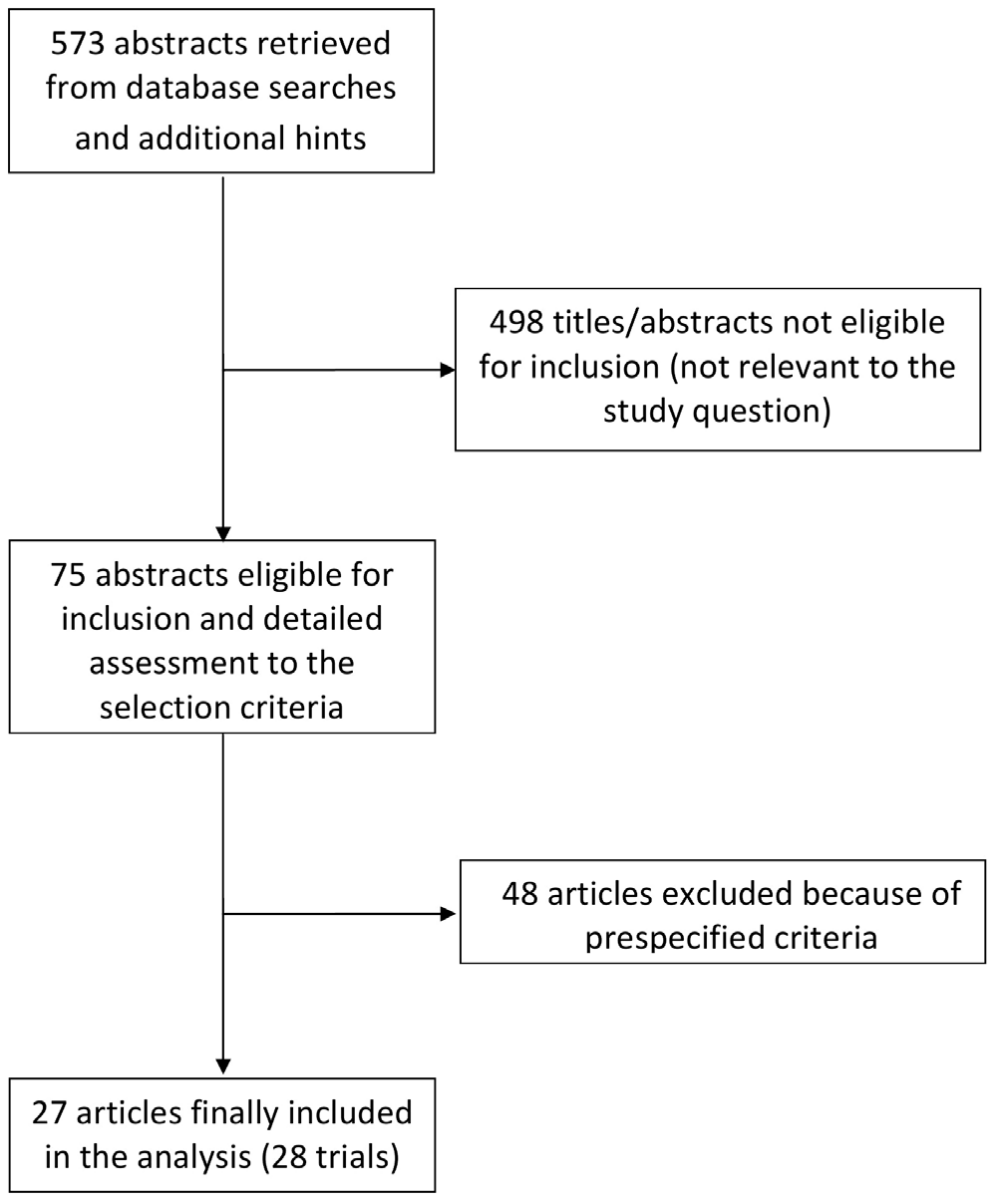
Flow diagram. The flow chart to select the final 27 manuscripts (28 trials).

Database searches, snowballing, and contacts with experts yielded a total of 573 articles. The flow chart to select the final 27 manuscripts (28 trials) [7]–[15], [21]–[38] is detailed in figure 1, with major exclusions available in the supplemental material (Texts S2 and S3).

The 27 included manuscripts randomized 3,648 patients (1,870 to dexmedetomidine and 1,778 to control) (tables 1 and 2). Clinical heterogeneity was mostly due to setting, control treatment, and follow-up duration. Indeed, 13 trials used dexmedetomidine in a general ICU setting [8], [12], [13], [26]–[29], [31]–[34], [37], ten in cardiac surgery ICU patients [7], [9], [10], [14], [15], [21], [24], [25], [30], [35], four in major non-cardiac surgery ICU patients [11], [22], [36], [38] and one after caesarean section-eclampsia admitted to ICU [23]. Different techniques of dexmedetomidine administration were used: in 18 trials the continuous infusion was preceded by a loading dose that was often 1 mcg/kg [13], [14], [17], [23], [24], [26], [28]–[30], [32], [33], [36] but that varied between 0.1 to 6 mcg/kg in other trials [11], [22], [25], [27], [30], [34], [37], [38]. In other 6 trials only continuous infusion was used and ranged between 0.1 to 2.5 mcg/kg/h [7]–[9], [15], [31], [35] while in one trial only the loading dose was used [10] and one trial gave no details [12]. Study quality appraisal indicated that trials were of medium quality (Table S1); in particular 12of them had a low risk of bias.

Six different comparators were identified: propofol in 11 study arms [8], [11], [17], [22], [24], [25], [27], [29], [34], [36], [38], midazolam in 10 arms [8], [12], [13], [15], [23], [25], [28], [30], [33], [34], placebo in 5 arms [9], [10], [14], [26], [37], morphine in 2 arms [7], [35], haloperidol [32] and lorazepam [31] in one study.

### Quantitative Data Synthesis

#### Effect of dexmedetomidine on ICU stay and time to extubation

**Figure 2:**
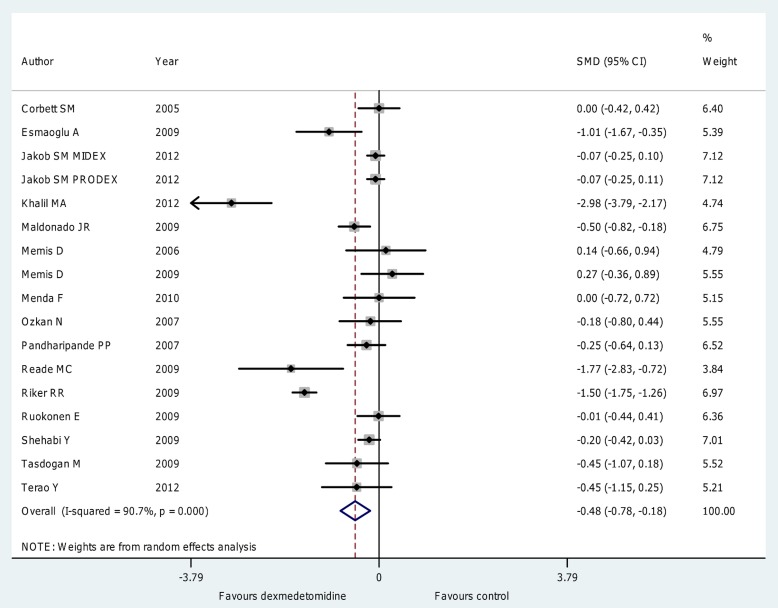
Forest plot for the length of ICU stay. Overall analysis showed that the use of dexmedetomidine was associated with a significant reduction in length of ICU stay (SMD = −0.48 [−0.78 to −0.18] , p for effect = 0.002, p for heterogeneity <0.001, I2 = 91% with 17 studies and 2,424 patients included). ICU = intensive care unit; CI = confidence interval; SMD = standardized mean difference; N = number; SD = standard deviation; Dex = dexmedetomidine.

Overall analysis (figure 2; figure S1) showed that the use of dexmedetomidine was associated with a significant reduction in length of ICU stay (WMD = −0.79 [−1.17 to −0.40] days, p for effect <0.001, p for heterogeneity<0.001,I^2^ = 93%, SMD = −0.48 [−0.78 to −0.18], p for effect  = 0.002, p for heterogeneity <0.001, I^2^ = 91%; with 17 studies and 2,424 patients included) with results confirmed when subanalyses were performed on studies including patients undergoing elective surgery (SMD = −0.60 [−1.05 to −0.15], p for effect = 0.008 with 8 studies included), in those including patients undergoing short term sedation (SMD = −0.45 [−0.81 to −0.09], p for effect = 0.02 with 11 studies included), in those including patients receiving a loading dose (SMD = −0.58 [−1.03 to −0.13], p for effect = 0.01 with 11 studies included) and in those receiving low (<0.7 µg kg−1 h−1) maintenance dose of dexmedetomidine (SMD = −0.62 [−1.04 to −0.20], p for effect = 0.004 with 10 studies included) as detailed in table 3.

**Table 3:** Sensitivity analyses of intensive care unit stay and time to extubation.

Outcome	Number of included trials	Dex patients	Controlpatients	SMD	95% CI	P for effect	P for heterogeneity	I^2^ (%)
Overall trials	28 trials (27 manuscripts)	1,870	1,778					
**ICU stay**	**17**	**1,274**	**1,150**	**−0.48**	**−0.78 to −0.18**	**0.002**	**<0.001**	**91**
-Postoperative elective surgery patients	8	373	372	−0.60	−1.05 to −0.15	0.008	<0.001	86
---- Cardiac surgery	6	337	336	−0.57	−1.11 to −0.03	0.04	<0.001	89
---- CABG surgery	4	103	106	−0.76	−1.94 to 0.42	0.2	<0.001	93
---- Non Cardiac surgery	2	36	36	−0.75	−1.23 to −0.27	0.002	0.3	23
-ICU patients (non elective postoperative)	9	901	778	−0.38	−0.82 to 0.06	0.09	<0.001	93
-excluding outlier studies [14], [32], [33]	13	1438	1427	−0.17	−0.29 to −0.05	0.005	0.15	28
- Long term sedation	6	849	726	−0.54	−1.09 to 0.008	0.053	<0.001	95
- Short term sedation	11	425	424	−0.45	−0.81 to −0.09	0.02	<0.001	82
- Daily interruption sedation	5	839	716	−0.38	−0.96 to 0.18	0.18	<0.001	96
- High maintenance dose	7	879	756	−0.31	−0.79 to 0.17	0.2	<0.001	95
- Low maintenance dose(<0.7 µg kg−1 h−1)	10	395	394	−0.62	−1.04 to −0.20	0.004	<0.001	85
- Loading dose	11	281	282	−0.58	−1.03 to −0.13	0.01	<0.001	83
- No loading dose	6	993	868	−0.36	−0.81 to 0.10	0.13	<0.001	96
- Loading dose and high maintenance dose	2	140	40	−0.09	−0.79 to 0.61	0.8	0.12	60
- SENSITIVITY (including only blinded studies)	8	891	768	−0.56	−1.09 to −0.04	0.04	<0.001	95
SENSITIVITY (including only low risk of bias studies)	10	1065	940	−0.44	−0.86 to −0.02	0.04	<0.001	94
SENSITIVITY (removing 1 study at time)	All 95% CIs of SDM<0 and p<0.05							
SMALL STUDY EFFECT (fixed model)				−0.34	−0.43 to −0.26	<0.001		
**Time to extubation**	**24**	**1,804**	**1,674**	**−0.39**	**−0.66 to −0.11**	**0.005**	**<0.001**	**93**
-Postoperative elective surgery patients	17	954	942	−0.31	−0.52 to −0.09	0.005	<0.001	77
---- Cardiac surgery	10	558	555	−0.42	−0.75 to −0.10	0.01	<0.001	83
---- CABG surgery	7	310	311	−0.59	−1.13 to −0.05	0.03	<0.001	89
---- Non Cardiac surgery (3 studies did not specify the operative setting)	4	76	76	−0.15	−0.47 to 0.17	0.4	0.8	0
-ICU patients (non elective postoperative)	7	850	732	−0.52	−1.25 to 0.21	0.16	<0.001	97
-excluding outlier studies [14], [32], [33]	20	995	993	−0.16	−0.26 to −0.05	0.003	0.04	39
- Long term sedation	6	830	712	−0.65	−1.44 to 0.15	0.11	<0.001	98
- Short term sedation	18	974	962	−0.28	−0.49 to −0.07	0.009	<0.001	76
- Daily interruption sedation	4	785	664	−0.69	−1.70 to 0.32	0.18	<0.001	99
- High maintenance dose	7	859	737	−0.42	−1.13 to 0.30	0.3	<0.001	76
- Low maintenance dose(<0.7 µg kg−1 h−1)	16	843	839	−0.30	−0.53 to −0.07	0.009	<0.001	76
- Loading dose	16	734	731	−0.23	−0.47 to 0.001	0.051	<0.001	75
- No loading dose	7	968	845	−0.60	−1.25 to 0.05	0.07	<0.001	97
- Loading dose and high maintenance dose	3	74	73	−0.08	−0.44 to 0.27	0.3	0.3	11
SENSITIVITY (including only blinded studies)	10	1241	1112	−0.56	−1.06 to −0.05	0.03	<0.001	97
SENSITIVITY (including only low risk of bias studies)	8	1023	899	−0.72	−1.34 to −0.10	0.02	<0.001	97
SENSITIVITY (removing 1 study at time)	All 95% CIs of SDM<0 and p<0.05							
SENSITIVITY(Jakob study [8] included as time on mechanical ventilation) *	24	1,804	1,674	−0.38	−0.66 to −0.10	0.007	<0.001	93
SMALL STUDY EFFECT (fixed model)				−0.31	−0.38 to 0.24	<0.001		

The overall analyses using weighted mean differences showed a reduction in intensive care unit stay of −0.79 [−1.17 to −0.40] days and a reduction in time to extubation of −2.74 [−3.80 to −1.65] hours in the dexmedetomidine group. It should be noted that the standard mean differences used in this table is not expressed in days or hours.

Dex: dexmedetomidine; SMD: standardized mean difference; CI: confidence interval; P: p-value; CABG: coronary artery bypass grafting; ICU: intensive care unit; NIV: non invasive ventilation.

duration of mechanical ventilation from randomization until patients were free of mechanical ventilation(including noninvasive).

**Figure 3:**
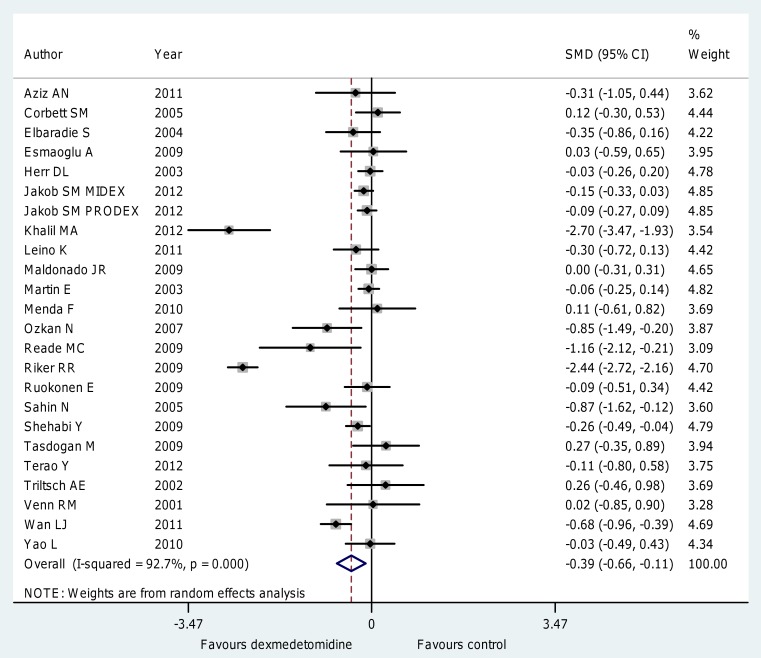
Forest plot for the time to extubation. Overall analysis showed that the use of dexmedetomidine was associated with a significant reduction of time to extubation (SMD = −0.39 [−0.66 to −0.11], p for effect = 0.005, p for heterogeneity <0.001, I2 = 93% with 24 studies and 3,478 patients included). CI = confidence interval; SMD = standardized mean difference; N = number; SD = standard deviation; Dex = dexmedetomidine.

The use of dexmedetomidine was also associated (figure 3; figure S2) with a significant reduction of time to extubation (WMD = −2.74 [−3.80 to −1.65] hours, p for effect <0.001, p for heterogeneity<0.001,I^2^ = 96%, SMD = −0.39 [−0.66 to −0.11], p for effect = 0.005, p for heterogeneity <0.001, I^2^ = 93% with 24 studies and 3,478 patients included). Further subanalyses, detailed in table 3, confirmed these findings in patients receiving short term sedation (SMD = −0.28 [−0.49 to −0.07], p for effect = 0.009 with 18 studies included), in those receiving a low (<0.7 µg kg−1 h−1) maintenance dose (SMD = −0.30 [−0.53 to −0.07], p for effect = 0.009 with 16 studies included) and in those undergoing elective surgery (SMD = −0.31 [−0.52 to −0.09], p for effect = 0.005 with 17 studies included) with most of the positive finding coming from the cardiac surgery setting (SMD = −0.42 [−0.75 to −0.10], p for effect = 0.01 with 10 studies included). The largest study [8] included in this meta-analysis was also the only one to report both median and mean values for mechanical ventilation. Since these data were skewed, we repeated the analyses including median instead of mean values and didn't find differences in pooled estimate results (SMD = −0.39, 95% CI −0.66 to −0.12, I-square = 93%).

Further subanalyses with the different comparators (propofol, midazolam, placebo and morphine) are detailed in supplemental material (TableS2 Table S3, Table S4 and Table S5) but were not informative with respect to ICU stay or time to extubation due to the paucity of trials included.

**Figure 4:**
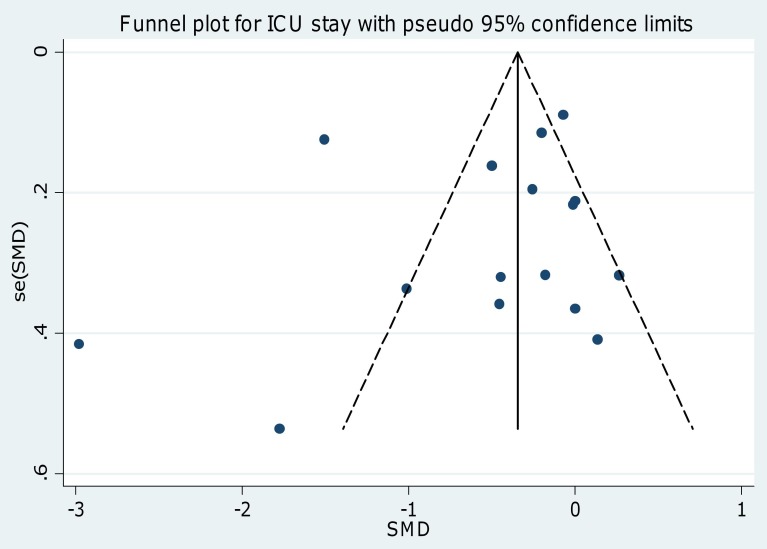
Funnel plot for the length of ICU stay. Visual inspection of funnel plots did not identify a skewed or asymmetrical shape for the co-primary endpoints. Quantitative evaluation did not suggest a presence of publication bias, as measured by the Egger's test (p = 0.4) and Peters' test (p = 0.6). ICU = intensive care unit; SE = standard error; SMD = standardized mean difference.

**Figure 5:**
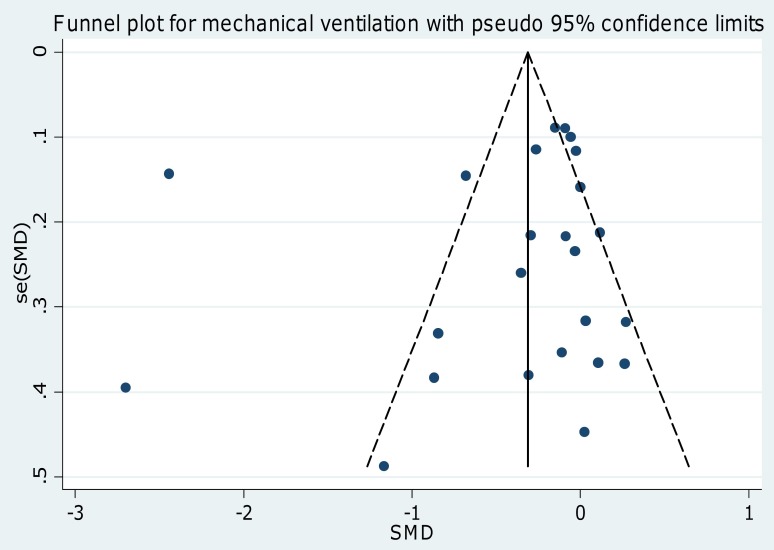
Funnel plot for the time to extubation. Visual inspection of funnel plots did not identify a skewed or asymmetrical shape for the co-primary endpoints. Quantitative evaluation did not suggest a presence of publication bias, as measured by the Egger's test (p = 0.5) and Peters' test (p = 0.9). SE = standard error; SMD =  standardized mean difference.

Visual inspection of funnel and scatter plots (figures 4 and 5; figures S3 and S4) did not identify a skewed or asymmetrical shape for the co-primary endpoints. Quantitative evaluation did not suggest a presence of publication bias, as measured by the Egger's test (p = 0.4 for the length of ICU stay and p = 0.5 for time to extubation) and Peters' test (p = 0.6 for the length of ICU stay and p = 0.9 for time to extubation). Since the funnel plots identified three outlier studies [14], [32], [33] we repeated the analyses removing them and found that the statistically significant difference in ICU stay and time to extubation was maintained (p = 0.005 and p = 0.003 respectively ) and the heterogeneity reduced (I^2^ = 28% and I^2^ = 39% respectively ) (table 3).

#### Effect of dexmedetomidine on rescue doses of analgesic drugs, incidence of bradycardia, hypotension and mortality

Rescue doses of sedative or analgesic drugs were required less in the dexmedetomidine patients (892/1,459 [61%] in the dexmedetomidine group versus 977/1,366 [72%] in the control arm, p = 0.01 with 14 studies included). A subanalysis showed that dexmedetomidine was associated with a significant reduction in the number of patients requiring rescue doses of analgesic drugs (691/927 [67%] in the dexmedetomidine group versus 624/802 [78%] in the control arm, RR = 0.80 [0.66 to 0.98], p = 0.03) with no differences in the number of patients requiring rescue doses of sedative drugs (271/532 [51%] in the dexmedetomidine group versus 353/564 [63%] in the control arm, p = 0.3) (Table 4).

**Table 4:** Secondary Outcomes.

Outcome	Number of included trials	Dex patients	Control patients	RR	95% CI	P for effect	P for heterogeneity	I^2^ (%)
Overall trials	28 trials (27 manuscripts)	1,870	1,778					
**Mortality**	20	200/1,499 [13%]	173/1,409 [12%]	1.00	0.84 to 1.21	0.9	0.9	0
**Hypotension**	19	424/1,389 [31%]	279/1,266 [22%]	1.27	1.00 to 1.61	0.052	<0.001	62
**Bradycardia**	17	220/1,374 [16%]	64/1,246 [5%]	2.43	1.88 to 3.14	<0.001	0.9	0
**Patients requiring rescue doses of either sedatives or analgesics**	14	892/1,459 [61%]	977/1,366 [72%]	0.81	0.70 to 0.95	0.01	<0.001	84
---requiring sedative drugs	8	271/532 [51%]	353/564 [63%]	0.84	0.62 to 1.14	0.3	<0.001	82
---requiring analgesic drugs	6	621/927 [67%]	624/802 [78%]	0.80	0.66 to 0.98	0.03	<0.001	88
**Number of patients completely comfortable**	3	112/253 [44%]	103/254 [40.6%]	1.07	0.49 to 2.49	0.9	0.003	82

Dex: dexmedetomidine; RR: relative risk; CI: confidence interval; P: p-value.

Dexmedetomidine was associated with an increased rate of bradycardia (220/1,374 [16%] in the dexmedetomidine group vs 64/1,246 [5%] in the control group, RR = 2.43[1.88 to 3.14], p for effect <0.001, p for heterogeneity = 0.9, I^2^ = 0% with 17 studies included) and with a trend towards an increased rate of hypotension (424/1,389 [31%] in the dexmedetomidine group vs 279/1,266 [22%] in the control group, RR = 1.27[1.00 to 1.61], p for effect 0.052, p for heterogeneity <0.001, I^2^ = 62% with 19 studies included) (Table 4).

No difference in mortality was recorded at the longest follow-up available (200/1,499 [13%] in the dexmedetomidine group vs 173/1,409 [12%] in the control group, RR = 1.00 [0.84 to 1.21], p for effect = 0.9 with 20 studies included). The univariate meta-regression of average follow-up against log-risk mortality showed no significant effects for time on mortality (n = 20, slope coefficient = −0.001 [−0.003 to 0.001], p = 0.31) (Table 4).

### Sensitivity analyses

Estimate results from both random and fixed effect models were extremely similar (table 3); hence we excluded a considerable small study effect. Sensitivity analyses performed by sequentially removing each study and reanalysing the remaining dataset (producing a new analysis for each study removed), did not determine major changes in direction or magnitude of statistical findings, confirming the pooled effect of each co-primary endpoints (all SWD<1) and the statistical significance (all p of effect <0 .05). Sensitivity analyses carried out with studies with low risk of bias confirmed the overall results of our work showing a reduction in length of ICU stay in dexmedetomidine versus control group (SMD = −0.44 [−0.86 to −0.02] p for effect = 0.04, p for heterogeneity <0.001, I^2^ = 94% with 10 studies and 2,005 patients included) and in time to extubation (SMD = −0.72 [−1.34 to −0.10], p for effect = 0.02, p for heterogeneity <0.001, I^2^ = 97% with 8 studies and 1,922 patients included). Sensitivity analyses carried out with blinded studies confirmed the overall results of our work showing a reduction in length of ICU stay in dexmedetomidine versus control group (SMD = −0.56 [−1.09 to −0.04], p for effect = 0.04, p for heterogeneity <0.001, I^2^ = 95% with 8 studies and 1,659patients included) and a reduction in time to extubation (SMD = −0.56 [−1.06 to 0.05], p for effect = 0.03, p for heterogeneity <0.001, I^2^ = 97% with 10 studies and 2,353 patients included).

## Discussion

Our meta-analysis confirmed that dexmedetomidine is associated with a reduction in ICU stay and suggested that it might reduce the time of extubation when compared to other sedative or hypnotic agent. Even if dexmedetomidine is associated with an increase in the risk of bradycardia and with a trend toward an increased risk of hypotension, no detrimental effects on mortality were detected.

The ideal sedative agent should provide anticipated, predictable effects, rapid onset, and quick recovery. It should be easy to administer with no adverse events, no interaction with other drugs, no accumulation of metabolites and no withdrawal effects at the end of infusion. Unluckily an ideal sedative agent that can suit the need of all patients does not yet exist.

Dexmedetomidine is one of the most recently released intravenous agents for sedation in the ICU, though the drug started to be investigated more than 20 years ago. It was introduced in clinical practice in the United States in 1999 while the European Medicine Agency authorised its use for all 27 European member states in September 2011. It is an alpha2-agonist and produces sedation acknowledged as “cooperative” or “arousable”, which is different from the sedation “clouding of consciousness” induced by drugs acting on GABA receptors, such as midazolam or propofol [39]. Tan and Ho, in a previous meta-analysis updated on December 2009 [6] reported that when dexmedetomidine was compared with alternative sedative agents it was associated with a statistically significant reduction in length of ICU stay, but not in duration of mechanical ventilation. We updated their findings on February 2013 identifying eight recently published manuscripts [7]–[14] and one trial that was not identified in their systematic search [15], thus increasing the number of patients by 50% (up to 3,648 overall randomized patients included in our meta-analysis) and providing more robust safety data. By adding more patients data we were able to show, for the first time in a meta-analysis, that dexmedetomidine increases the rate of bradycardia when all trials are pooled together and also shows a trend towards an increase rate of hypotension. However, these side effects were not associated with differences in mortality (200/1499 [13%] in the dexmedetomidine group vs 173/1409 [12%] in the control group, p = 0.9 with 20 studies included).

Dexmedetomidine decreases sympathetic nervous system activity and is therefore associated with an increase in cardiovascular adverse events. These effects may be most pronounced in patients with decreased autonomic nervous system response such as the elderly, diabetic patients, patients with chronic hypertension or severe cardiac disease such as valve stenosis or regurgitation, advanced heart block, severe coronary artery disease, or in patients who are already hypotensive and/or hypovolemic [40]. Therefore, in patients who depend on a high level of sympathetic tone or in patients with reduced myocardial function who cannot tolerate the decrease in sympathetic tone, loading doses of dexmedetomidine should be avoided. On the other side, the characteristics of dexmedetomidine to provide an ongoing sedation and sympathetic block could be beneficial in reducing early postoperative ischemic events in high-risk patients [41]–[42].

Intravenous administration of dexmedetomidine exhibits the following pharmacokinetic parameters: a rapid distribution phase with an half-life (t 1/2 α) of 6 min, a terminal elimination half-life (t 1/2 β) of 2 hours, and a steady-state volume of distribution (Vss) of 118 litres. It presents linear kinetics when infused in the range of 0.2–0.7 µg/kg/h for no more than 24 hours and undergoes almost complete biotransformation through direct glucuronidation and cytochrome P450 metabolism. Consequently it can accumulate in patients who are on P450 enzyme inhibitors, some of which are commonly used in ICU. Metabolites of biotransformation are excreted in the urine (95%) and faeces [43].

### Limitations

We acknowledge that this study has several limitations. The quality of the included studies is not high since only 13 of them were blind. Moreover we noted high heterogeneity between the included studies. The heterogeneity remained when sensitivity analyses on studies with low risk of bias where performed. It was abolished only removing three outliers studies cited above. Nonetheless we excluded the possible influence of small-study effects on the results of our meta-analysis comparing the fixed- and random-effects estimates of the treatment effect (table 3). The overall reduction in ICU stay and time to extubation may appear clinically modest, but it should be acknowledged that the largest study [8] had very conservative imputation rules (to worst outcome) and this might have softened our results.

## Conclusions

Dexmedetomidine for sedation in mechanically ventilated critically ill adult patients seems to help to reduce time to extubation and ICU stay. The known side effects (increased incidence of bradycardia and a trend toward an increased risk of hypotension) had no effect on the overall mortality in this meta-analysis of all the RCTs published so far.

Larger, multicentre, randomized clinical trials, especially in long term sedated patients requiring mechanical ventilation, would be welcome to confirm these findings.

## Supporting Information

Checklist S1 **PRISMA checklist.**
(DOC)

Figure S1 **Forest plot for the length of ICU stay using standard mean difference (days) instead of weighted mean difference (absolute value with no units of measurement).** Overall analysis showed that the use of dexmedetomidine was associated with a significant reduction in length of ICU stay (SMD = −0.48 [−0.78 to −0.18], p for effect = 0.002, p for heterogeneity <0.001, I2 = 91% with 17 studies and 2,424 patients included). ICU = intensive care unit; CI = confidence interval; SMD = standardized mean difference; N = number; SD = standard deviation.
(TIF)

Figure S2 **Forest plot for the time to extubation using standard mean difference (days) instead of weighted mean difference (absolute value with no units of measurement)..** Overall analysis showed that the use of dexmedetomidine was associated with a significant reduction of time to extubation (SMD = −0.39 [−0.66 to −0.11], p for effect = 0.005, p for heterogeneity <0.001, I2 = 93% with 24 studies and 3,478 patients included). CI = confidence interval; SMD =  standardized mean difference; N = number; SD = standard deviation
(TIF)

Figure S3 **Scatter plot for ICU stay**
(TIF)

Figure S4 **Scatter plot for time to extubation**
(TIF)

Table S1 **Methodological quality summary: review authors' judgments about each methodological quality item for each included study.**
(DOCX)

Table S2 **Subanalysis with propofol as comparator drug**
(DOCX)

Table S3 **Subanalysis with midazolam as comparator drug**
(DOCX)

Table S4 **Subanalysis with morphine as comparator drug**
(DOCX)

Table S5 **Subanalysis with placebo as comparator drug**
(DOCX)

Text S1 **Full PubMed search strategy**
(DOCX)

Text S2 **Major exclusions**
(DOCX)

Text S3 **References of the excluded studies**
(DOCX)
