# Correction: Dexmedetomidine as a Sedative Agent in Critically Ill Patients: A Meta-Analysis of Randomized Controlled Trials

**DOI:** 10.1371/annotation/3f0f682b-f8aa-4e9c-9d60-8aac54bb4c22

**Published:** 2014-01-09

**Authors:** Laura Pasin, Teresa Greco, Paolo Feltracco, Annalisa Vittorio, Caetano Nigro Neto, Luca Cabrini, Giovanni Landoni, Gabriele Finco, Alberto Zangrillo

Formatting errors were introduced into Table 3 while preparing this article for publication. The headings in the row "Outcome" were erroneously shifted. Please view the corrected Table 3 here: http://plosone.org/corrections/pone.0082913.t003.cn.docx

