# Supplementary figures and images for: Dexmedetomidine as a Sedative Agent in Critically Ill Patients: A Meta-Analysis of Randomized Controlled Trials
Source: PLoS One. 2013 Dec 31;8(12):e82913. doi: 10.1371/journal.pone.0082913 (PMC3877008; doi:10.1371/journal.pone.0082913)

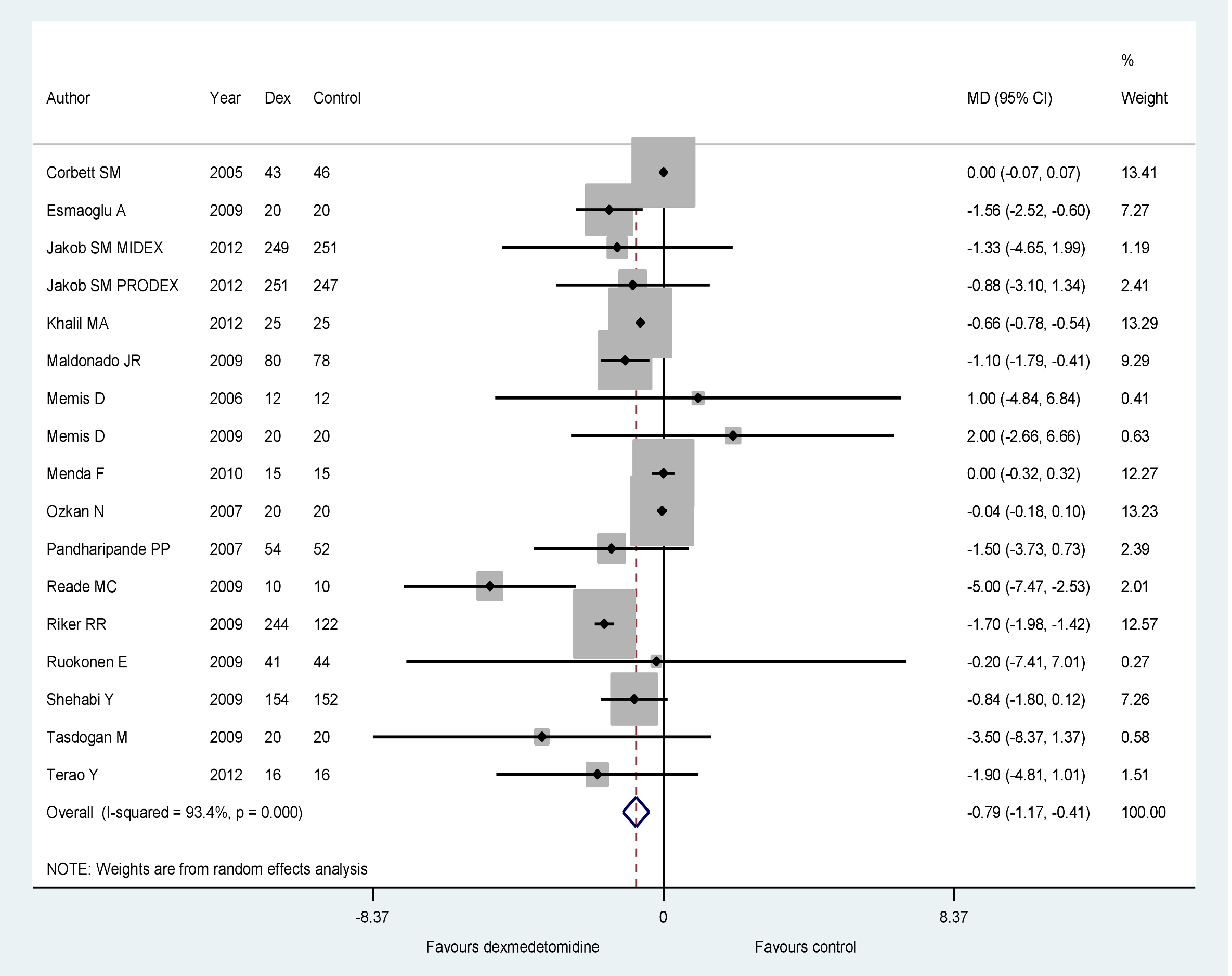

Supplement: Figure S1 — Forest plot for the length of ICU stay using standard mean difference (days) instead of weighted mean difference (absolute value with no units of measurement). Overall analysis showed that the use of dexmedetomidine was associated with a significant reduction in length of ICU stay (SMD = −0.48 [−0.78 to −0.18], p for effect = 0.002, p for heterogeneity <0.001, I2 = 91% with 17 studies and 2,424 patients included). ICU = intensive care unit; CI = confidence interval; SMD = standardized mean difference; N = number; SD = standard deviation. (TIF) [file pone.0082913.s002.tif]

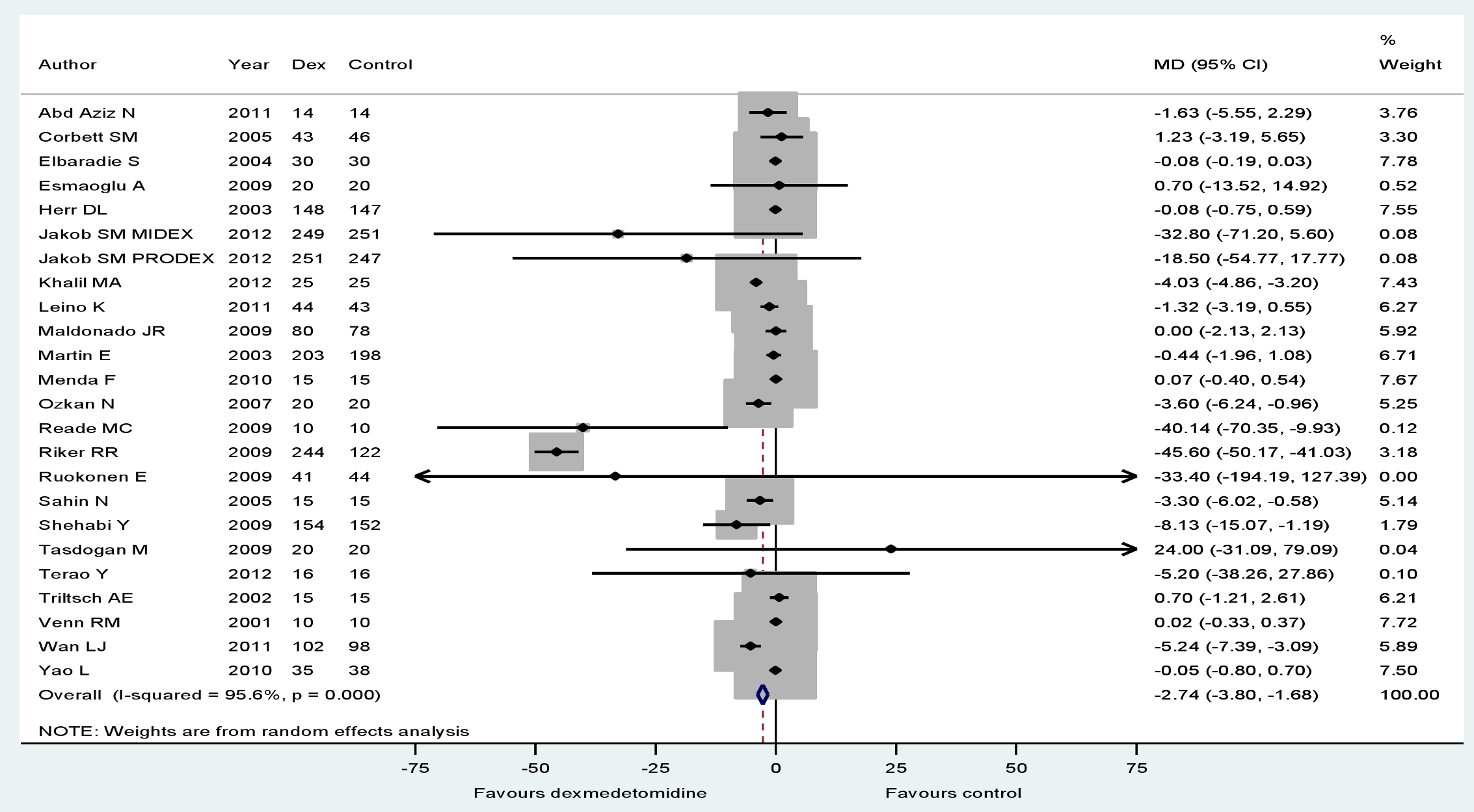

Supplement: Figure S2 — Forest plot for the time to extubation using standard mean difference (days) instead of weighted mean difference (absolute value with no units of measurement).. Overall analysis showed that the use of dexmedetomidine was associated with a significant reduction of time to extubation (SMD = −0.39 [−0.66 to −0.11], p for effect = 0.005, p for heterogeneity <0.001, I2 = 93% with 24 studies and 3,478 patients included). CI = confidence interval; SMD = standardized mean difference; N = number; SD = standard deviation (TIF) [file pone.0082913.s003.tif]

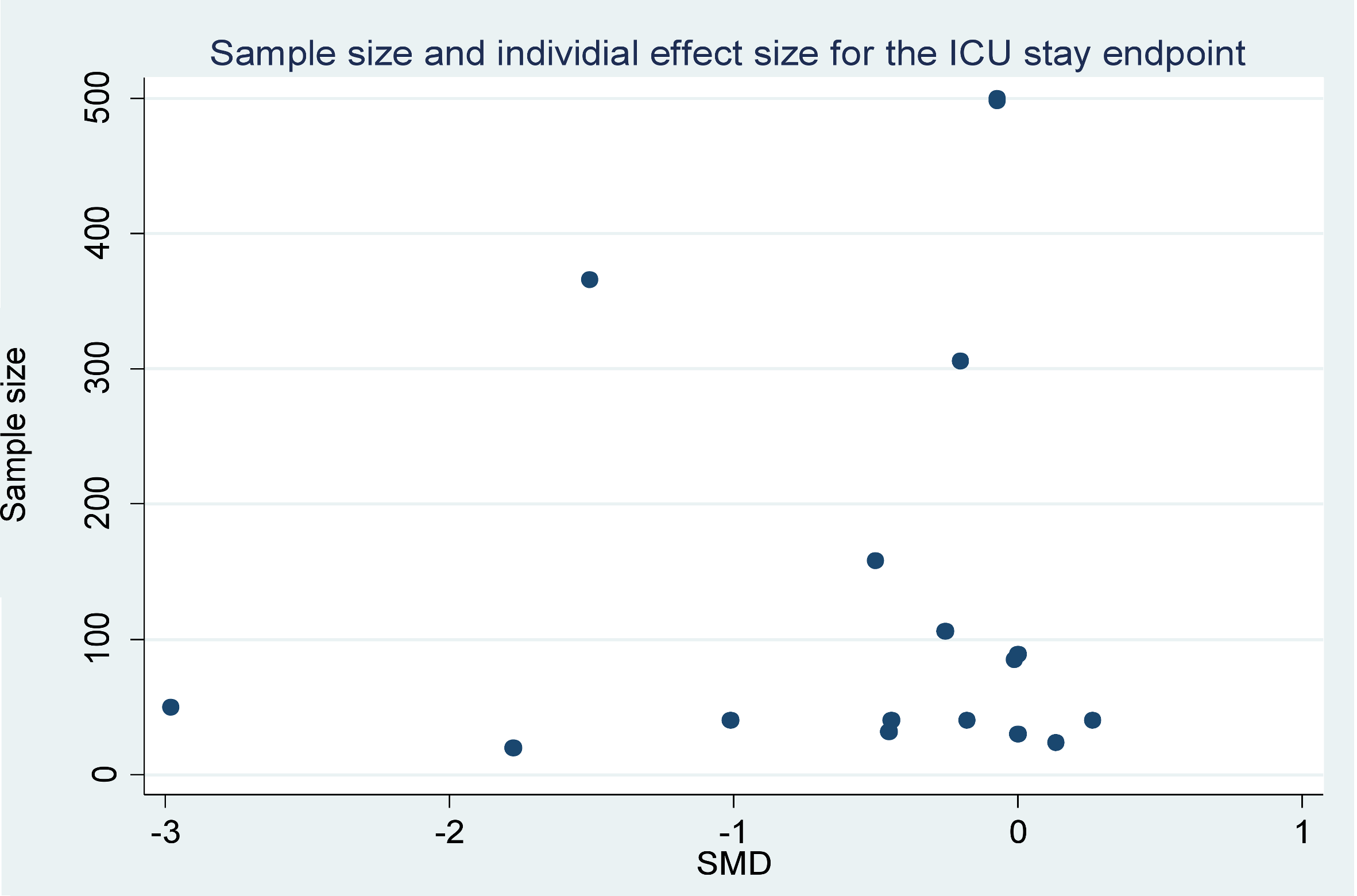

Supplement: Figure S3 — Scatter plot for ICU stay (TIF) [file pone.0082913.s004.tif]

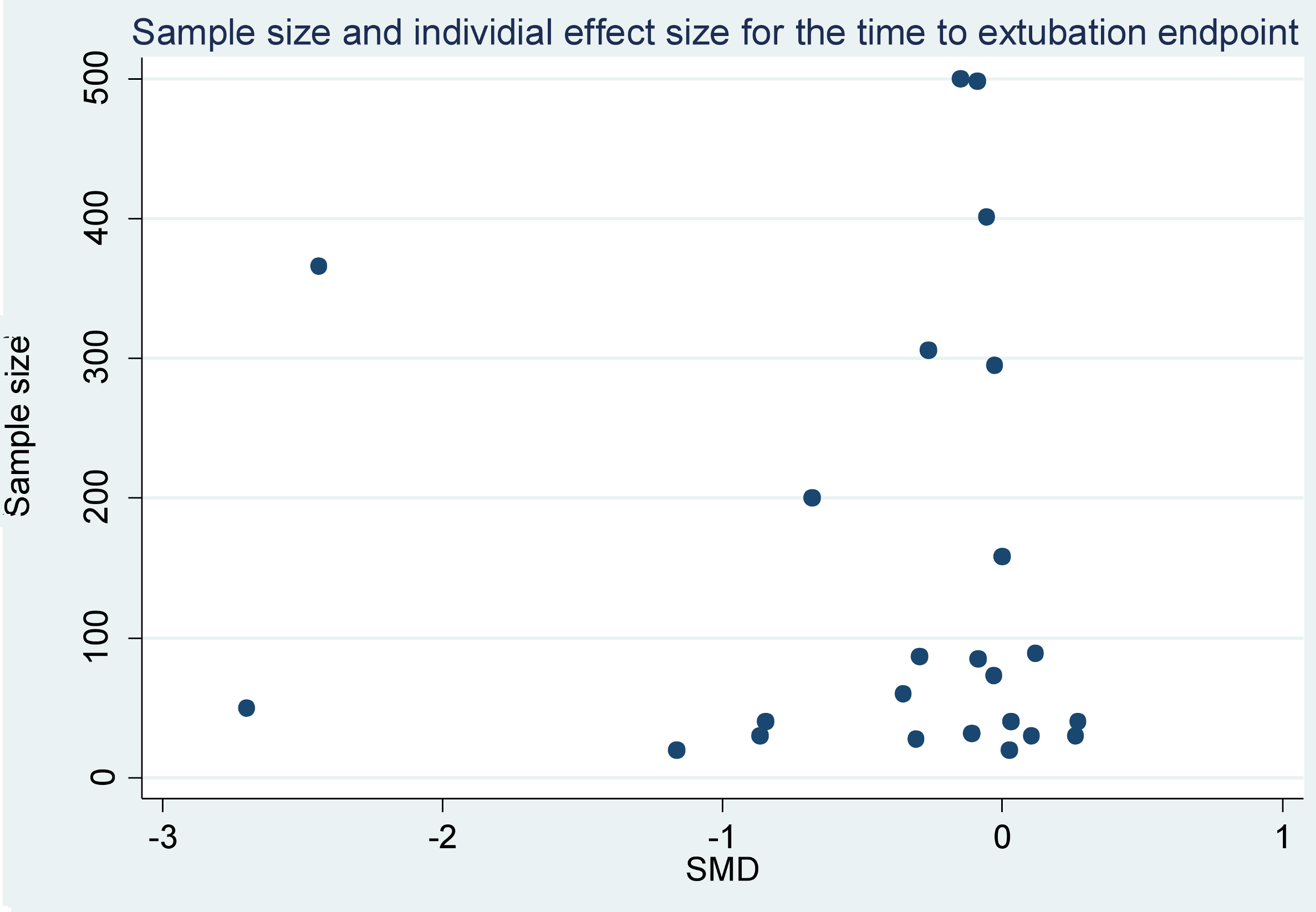

Supplement: Figure S4 — Scatter plot for time to extubation (TIF) [file pone.0082913.s005.tif]
