# Supplementary material for: Dexmedetomidine as a Sedative Agent in Critically Ill Patients: A Meta-Analysis of Randomized Controlled Trials
Source: PLoS One. 2013 Dec 31;8(12):e82913. doi: 10.1371/journal.pone.0082913 (PMC3877008; doi:10.1371/journal.pone.0082913)
Supplement: Table S1 — Methodological quality summary: review authors' judgments about each methodological quality item for each included study. (DOCX) [file pone.0082913.s006.docx]

| **TRIAL** | **ENTRY** | **JUDGMENT** | **DESCRIPTION** |
| --- | --- | --- | --- |
| Aziz NA [7] | Adequate sequence generation? | Unclear | The manuscript doesn't report the sequence generation method. |
|  | Allocation concealment? | Yes | Quote: "were randomly assigned using random envelope technique to receive either dexmedetomidine or morphine |
|  | Blinding of participants and personnel? | No | open-label trial |
|  | Blinding of outcome assesssment? | No | open-label trial |
|  | Complete outcome data addressed? | Unclear | Statistical analysis missing |
|  | Free of selective reporting? | Unclear | Statistical analysis missing |
|  | Free of other bias? | Yes | No fundings or conflicts of interest |
|  | OVERALL RISK OF BIAS | Moderate |  |
| Corbett SM [21] | Adequate sequence generation? | Unclear | The manuscript doesn't report the sequence generation method. |
|  | Allocation concealment? | Yes | Allocation concealment adequate |
|  | Blinding of participants and personnel? | No | The manuscript doesn't specify the type of blinding. We assume this is an open label study. |
|  | Blinding of outcome assesssment? | No | The manuscript doesn't specify the type of blinding. We assume this is an open label study. |
|  | Complete outcome data addressed? | Yes | analysis by intention to treat, all completed the study |
|  | Free of selective reporting? | Yes |  |
|  | Free of other bias? | Yes | Supported, in part, by the Society of Critical Care Medicine, Clinical Pharmacy and Pharmacology Section, Ortho-Biotech Fellowship Grant, and departmental funds. |
|  | OVERALL RISK OF BIAS | Moderate |  |
| Elbaradie S [22] | Adequate sequence generation? | No | patients were selected randomly using a toss into two equal groups |
|  | Allocation concealment? | No | single blinded |
|  | Blinding of participants and personnel? | No | single blinded |
|  | Blinding of outcome assesssment? | No | patient-blinded study |
|  | Complete outcome data addressed? | Yes | analysis by intention to treat, all completed the study |
|  | Free of selective reporting? | Yes |  |
|  | Free of other bias? | Unclear | Fundings or conflicts of interest not declared |
|  | OVERALL RISK OF BIAS | High |  |
| Esmaoglu A [23] | Adequate sequence generation? | No | The patients were randomly divided into 2 groups using coin toss. |
|  | Allocation concealment? | No | Allocation concealment inadequate |
|  | Blinding of participants and personnel? | No | The manuscript doesn't specify the type of blinding. We assume this is an open label study. |
|  | Blinding of outcome assesssment? | No | The manuscript doesn't specify the type of bliding. We assume this is an open label study. |
|  | Complete outcome data addressed? | Yes | analysis by intention to treat, all completed the study |
|  | Free of selective reporting? | Yes |  |
|  | Free of other bias? | Unclear | Fundings or conflicts of interest not declared |
|  | OVERALL RISK OF BIAS | High |  |
| Herr DL [24] | Adequate sequence generation? | Unclear | Quote : "Investigators did not know the randomization" block size. |
|  | Allocation concealment? | Yes | sealed envelopes |
|  | Blinding of participants and personnel? | No | unclear |
|  | Blinding of outcome assesssment? | No | unclear |
|  | Complete outcome data addressed? | Yes | analysis by intention to treat, all completed the study |
|  | Free of selective reporting? | Yes |  |
|  | Free of other bias? | No | Supported by Abbott Laboratories. |
|  | OVERALL RISK OF BIAS | Moderate |  |
| Jakob SM MIDEX [8] | Adequate sequence generation? | Yes | stratified for study center in blocks of 4 |
|  | Allocation concealment? | Yes | Treatments were administered in a double dummy design |
|  | Blinding of participants and personnel? | Yes | double-blind |
|  | Blinding of outcome assesssment? | Yes | double blind |
|  | Complete outcome data addressed? | No | analysis by intention to treat, 8% did not complete the study |
|  | Free of selective reporting? | Yes |  |
|  | Free of other bias? | No | sponsored by Orion Pharma |
|  | OVERALL RISK OF BIAS | Low |  |
| Jakob SM PRODEX [8] | Adequate sequence generation? | Yes | stratified for study center in blocks of 4 |
|  | Allocation concealment? | Yes | Treatments were administered in a double dummy design |
|  | Blinding of participants and personnel? | Yes | double-blind |
|  | Blinding of outcome assesssment? | Yes | double blind |
|  | Complete outcome data addressed? | No | analysis by intention to treat, 8.2% did not complete the study |
|  | Free of selective reporting? | Yes |  |
|  | Free of other bias? | No | sponsored by Orion Pharma |
|  | OVERALL RISK OF BIAS | Low |  |
| Khalil MA [14] | Adequate sequence generation? | Yes | patients were allocated using a randomized number table |
|  | Allocation concealment? | Yes |  |
|  | Blinding of participants and personnel? | Yes | Blinding of the study was assured by investigators who were not related to the study including anesthesia and ICU. |
|  | Blinding of outcome assesssment? | Unclear |  |
|  | Complete outcome data addressed? | Yes |  |
|  | Free of selective reporting? | Yes |  |
|  | Free of other bias? | Yes | No fundings or conflicts of interest |
|  | OVERALL RISK OF BIAS | Low |  |
| Leino K [9] | Adequate sequence generation? | Yes | Randomization was performed by an independent statistician using random permuted blocks of 10 patients within each center |
|  | Allocation concealment? | Yes | Quote : "were provided with indistinguishable ampoules of dexmedetomidine and placebo to be allotted in numerical order to consecutive patients enrolled by study physicians". |
|  | Blinding of participants and personnel? | Yes | All patients, personnel and investigators including the persons responsible for data management and statistics were blinded to the treatment assignment |
|  | Blinding of outcome assesssment? | Yes |  |
|  | Complete outcome data addressed? | Yes | Both PP e ITT analysis |
|  | Free of selective reporting? | Yes |  |
|  | Free of other bias? | Unclear | This study was funded by Orion-Pharma (Finland) |
|  | OVERALL RISK OF BIAS | Low |  |
| Maldonado JR [25] | Adequate sequence generation? | Unclear | Random drowing; blocking |
|  | Allocation concealment? | No |  |
|  | Blinding of participants and personnel? | No | open-label |
|  | Blinding of outcome assesssment? | No |  |
|  | Complete outcome data addressed? | No | analysis by intention to treat, 24% did not complete the study |
|  | Free of selective reporting? | Yes |  |
|  | Free of other bias? | Unclear | Fundings or conflicts of interest not declared |
|  | OVERALL RISK OF BIAS | High |  |
| Martin E [26] | Adequate sequence generation? | Unclear | The manuscript doesn't report the seqecence generation method. |
|  | Allocation concealment? | Yes | Quote: "Both solutions were identical in appearance and viscosity" |
|  | Blinding of participants and personnel? | Yes | double blind |
|  | Blinding of outcome assesssment? | Yes | double blind |
|  | Complete outcome data addressed? | No | analysis by intention to treat, 2.5% did not complete the study |
|  | Free of selective reporting? | Yes |  |
|  | Free of other bias? | No | Sponsored by Abbott |
|  | OVERALL RISK OF BIAS | Moderate |  |
| Memis D [27] | Adequate sequence generation? | Yes | computer-steered permuted block design |
|  | Allocation concealment? | Yes | drug solution and infusion set were covered with foil |
|  | Blinding of participants and personnel? | Yes | double blind |
|  | Blinding of outcome assesssment? | Yes | double blind |
|  | Complete outcome data addressed? | Yes | analysis by intention to treat, all completed the study |
|  | Free of selective reporting? | Yes |  |
|  | Free of other bias? | Unclear | Fundings or conflicts of interest not declared |
|  | OVERALL RISK OF BIAS | Low |  |
| Memis D [28]^a^ | Adequate sequence generation? | Unclear | The manuscript doesn't report the seqecence generation method. |
|  | Allocation concealment? | Yes | sealed envelopes |
|  | Blinding of participants and personnel? | Unclear | unclear blinding |
|  | Blinding of outcome assesssment? | Unclear | unclear blinding |
|  | Complete outcome data addressed? | Yes | analysis by intention to treat, all completed the study |
|  | Free of selective reporting? | Yes |  |
|  | Free of other bias? | Unclear | Fundings or conflicts of interest not declared |
|  | OVERALL RISK OF BIAS | Low |  |
| Memis D [29] | Adequate sequence generation? | Yes | computer generated |
|  | Allocation concealment? | Yes | sealed envelopes |
|  | Blinding of participants and personnel? | No | unblinded |
|  | Blinding of outcome assesssment? | No | unblinded |
|  | Complete outcome data addressed? | Yes | analysis by intention to treat, all completed the study |
|  | Free of selective reporting? | Yes |  |
|  | Free of other bias? | Yes | supported by the University of Trakya, Edirne, Turkey |
|  | OVERALL RISK OF BIAS | Low |  |
| Menda F [10] | Adequate sequence generation? | Unclear | The manuscript doesn't report the seqecence generation method. |
|  | Allocation concealment? | Yes | closed envelope method |
|  | Blinding of participants and personnel? | Yes | double blind |
|  | Blinding of outcome assesssment? | Yes | double blind |
|  | Complete outcome data addressed? | Yes | analysis by intention to treat, all completed the study |
|  | Free of selective reporting? | Yes |  |
|  | Free of other bias? | Yes | No fundings or conflicts of interest |
|  | OVERALL RISK OF BIAS | Low |  |
| Ozkan N [30] ^b^ | Adequate sequence generation? | Unclear | The manuscript doesn't report the seqecence generation method. |
|  | Allocation concealment? | Unclear | Allocation concealment unclear |
|  | Blinding of participants and personnel? | No | unblinded |
|  | Blinding of outcome assesssment? | No | unblinded |
|  | Complete outcome data addressed? | Yes | analysis by intention to treat, all completed the study |
|  | Free of selective reporting? | Yes |  |
|  | Free of other bias? | Unclear | Fundings or conflicts of interest not declared |
|  | OVERALL RISK OF BIAS | Moderate |  |
| Pandharipande PP [31] | Adequate sequence generation? | Yes | computer-generated, permuted block randomization |
|  | Allocation concealment? | Yes | clear bags |
|  | Blinding of participants and personnel? | Yes | double blind |
|  | Blinding of outcome assesssment? | Yes | double blind |
|  | Complete outcome data addressed? | Yes | intention to treat, 2.8% did not complete the study |
|  | Free of selective reporting? | Yes |  |
|  | Free of other bias? | Yes | supported by Hospira Inc |
|  | OVERALL RISK OF BIAS | Low |  |
| Reade MC [32] | Adequate sequence generation? | Yes | computer generated |
|  | Allocation concealment? | No | unblinded |
|  | Blinding of participants and personnel? | No | unblinded |
|  | Blinding of outcome assesssment? | No | unblinded |
|  | Complete outcome data addressed? | Yes | analysis by intention to treat, all completed the study |
|  | Free of selective reporting? | Yes |  |
|  | Free of other bias? | Yes | in part supported by grants from the Australian College of Critical Care Nurses and the Australian and New Zealand College of Anaesthetists. Dexmedetomidine was supplied free of charge by the manufacturer, Hospira, who had no other involvement in the study. |
|  | OVERALL RISK OF BIAS | Moderate |  |
| Riker RR [33] | Adequate sequence generation? | Yes | computer-generated |
|  | Allocation concealment? | Yes | Adequate |
|  | Blinding of participants and personnel? | Yes | double blind |
|  | Blinding of outcome assesssment? | Yes | double blind |
|  | Complete outcome data addressed? | No | analysis not by intention to treat, 2.4% did not complete the study |
|  | Free of selective reporting? | Yes |  |
|  | Free of other bias? | Yes | funded by Hospira |
|  | OVERALL RISK OF BIAS | Low |  |
| Ruokonen E [34) | Adequate sequence generation? | Unclear | The manuscript doesn't report the seqecence generation method. |
|  | Allocation concealment? | Unclear | Allocation concealment unclear |
|  | Blinding of participants and personnel? | Yes | double blind |
|  | Blinding of outcome assesssment? | Yes | double blind |
|  | Complete outcome data addressed? | Yes | analysis not by intention to treat, all completed the study |
|  | Free of selective reporting? | Yes |  |
|  | Free of other bias? | Yes | funded by Orion Pharma |
|  | OVERALL RISK OF BIAS | Low |  |
| Sahin N [15]^b^ | Adequate sequence generation? | Unclear | The manuscript doesn't report the sequence generation method. |
|  | Allocation concealment? | Unclear | Allocation concealment unclear |
|  | Blinding of participants and personnel? | No | The manuscript doesn't specify the type of blinding. We assume this is an open label study. |
|  | Blinding of outcome assesssment? | No | The manuscript doesn't specify the type of blinding. We assume this is an open label study. |
|  | Complete outcome data addressed? | Unclear | Available as abstract only |
|  | Free of selective reporting? | Unclear | Available as abstract only |
|  | Free of other bias? | Unclear | Fundings or conflicts of interest not declared |
|  | OVERALL RISK OF BIAS | High |  |
| Shehabi Y [35] | Adequate sequence generation? | Yes | computer generated |
|  | Allocation concealment? | Yes | Adequate |
|  | Blinding of participants and personnel? | Yes | double blind |
|  | Blinding of outcome assesssment? | Yes | double blind |
|  | Complete outcome data addressed? | No | analysis by intention to treat, 2.3% did not complete the study |
|  | Free of selective reporting? | Yes |  |
|  | Free of other bias? | Unclear | drug funded by Hospira |
|  | OVERALL RISK OF BIAS | Low |  |
| Tasdogan M [36] | Adequate sequence generation? | Unclear | The manuscript doesn't report the sequence generation method. |
|  | Allocation concealment? | Yes | Adequate |
|  | Blinding of participants and personnel? | No | unblinded |
|  | Blinding of outcome assesssment? | No | unblinded |
|  | Complete outcome data addressed? | Yes | analysis not by intention to treat, all completed the study |
|  | Free of selective reporting? | Yes |  |
|  | Free of other bias? | Yes | funded by University of Trakya Research Grant |
|  | OVERALL RISK OF BIAS | Moderate |  |
| Terao Y [11] | Adequate sequence generation? | Unclear | The manuscript doesn't report the sequence generation method. |
|  | Allocation concealment? | Yes | sealed envelope |
|  | Blinding of participants and personnel? | No | unblinded |
|  | Blinding of outcome assesssment? | No | unblinded |
|  | Complete outcome data addressed? | Yes | analysis by intention to treat, all completed the study |
|  | Free of selective reporting? | Yes |  |
|  | Free of other bias? | Yes | supported, in part, by research funds of the Japan Labor Health and Welfare Organization to promote function. |
|  | OVERALL RISK OF BIAS | Moderate |  |
| Triltsch AE [37] | Adequate sequence generation? | Unclear | The manuscript doesn't report the sequence generation method. |
|  | Allocation concealment? | Unclear | Allocation concealment unclear |
|  | Blinding of participants and personnel? | Yes | double blind |
|  | Blinding of outcome assesssment? | Yes | double blind |
|  | Complete outcome data addressed? | No | analysis by intention to treat, 6.7% did not complete the study |
|  | Free of selective reporting? | Yes |  |
|  | Free of other bias? | No | funded in part by Abbott Laboratories |
|  | OVERALL RISK OF BIAS | Moderate |  |
| Venn RM [38] | Adequate sequence generation? | Unclear | The manuscript doesn't report the sequence generation method. |
|  | Allocation concealment? | Yes | Adequate |
|  | Blinding of participants and personnel? | No | unblinded |
|  | Blinding of outcome assesssment? | No | unblinded |
|  | Complete outcome data addressed? | Yes | analysis by intention to treat, all completed the study |
|  | Free of selective reporting? | Yes |  |
|  | Free of other bias? | Yes | funded by Abbott Laboratories |
|  | OVERALL RISK OF BIAS | Moderate |  |
| Wan LJ [12] ^b^ | Adequate sequence generation? | Yes | Numerical table method |
|  | Allocation concealment? | Unclear | Allocation concealment unclear |
|  | Blinding of participants and personnel? | No | The manuscript doesn't specify the type of blinding. We assume this is an open label study. |
|  | Blinding of outcome assesssment? | No | The manuscript doesn't specify the type of blinding. We assume this is an open label study. |
|  | Complete outcome data addressed? | Unclear | Available as abstract only |
|  | Free of selective reporting? | Unclear |  |
|  | Free of other bias? | Unclear | Fundings or conflicts of interest not declared |
|  | OVERALL RISK OF BIAS | High |  |
| Yao L [13] ^b^ | Adequate sequence generation? | Unclear | The manuscript doesn't report the sequence generation method. |
|  | Allocation concealment? | Unclear | Allocation concealment unclear |
|  | Blinding of participants and personnel? | No | The manuscript doesn't specify the type of blinding. We assume this is an open label study. |
|  | Blinding of outcome assesssment? | No | The manuscript doesn't specify the type of blinding. We assume this is an open label study. |
|  | Complete outcome data addressed? | Unclear | Available as abstract only |
|  | Free of selective reporting? | Unclear |  |
|  | Free of other bias? | Unclear | Fundings or conflicts of interest not declared |
|  | OVERALL RISK OF BIAS | High |  |

^a^ Letter to editor

^b^ Study published as abstract only
