# Supplementary material for: Dexmedetomidine as a Sedative Agent in Critically Ill Patients: A Meta-Analysis of Randomized Controlled Trials
Source: PLoS One. 2013 Dec 31;8(12):e82913. doi: 10.1371/journal.pone.0082913 (PMC3877008; doi:10.1371/journal.pone.0082913)
Supplement: Table S2 — Subanalysis with propofol as comparator drug (DOCX) [file pone.0082913.s007.docx]

| **Outcome** Comparator PROPOFOL | **Number of included trials** | **Dex**  **patients** | **Control**  **patients** | **SMD** | **95% CI** | **P for effect** | **P for heterogeneity** | **I^2^ (%)** |
| --- | --- | --- | --- | --- | --- | --- | --- | --- |
| **ICU stay** |  |  |  |  |  |  |  |  |
| **Overall trials** | **5 trials** | **346** | **345** | **-0.06** | **-0.21 to 0.09** | **0.5** | **0.6** | **0** |
| - Long term sedation | 1 | 251 | 247 | - | - | - | - | - |
| - Short term sedation | 4 | 95 | 98 | -0.02 | -0.30 to 0.27 | 0.9 | 0.4 | 0 |
| - Daily interruption sedation | 1 | 251 | 247 | - | - | - | - | - |
| - High maintenance doses dex | 3 | 291 | 287 | -0.08 | -0.32 to 0.17 | 0.6 | 0.3 | 20 |
| - No high maintenance doses dex | 2 | 55 | 58 | 0.03 | -0.34 to 0.40 | 0.9 | 0.8 | 0 |
| - Loading dose dex | 4 | 95 | 98 | -0.02 | -0.30 to 0.27 | 0.9 | 0.4 | 0 |
| - No loading dose dex | 1 | 251 | 247 | - | - | - | - | - |
| - High and loading doses dex | 1 | 251 | 247 | - | - | - | - | - |
| - Blind | 2 | 263 | 259 | -0.06 | -0.23 to 0.41 | 0.5 | 0.6 | 0 |
| - CABG | 1 | 43 | 46 | - | - | - | - | - |
| - Low risk of bias studies | 3 | 283 | 279 | -0.04 | -0.20 to 0.13 | 0.7 | 0.5 | 0 |
|  |  |  |  |  |  |  |  |  |
| **Time to extubation** |  |  |  |  |  |  |  |  |
| **Overall trials** | **6 trials** | **502** | **500** | **-0.05** | **-0.18 to 0.07** | **0.4** | **0.6** | **0** |
| - Long term sedation | 1 | 251 | 247 | - | - | - | - | - |
| - Short term sedation | 5 | 251 | 253 | -0.02 | -0.19 to 0.16 | 0.9 | 0.6 | 0 |
| - Daily interruption sedation | 1 | 251 | 247 | - | - | - | - | - |
| - High maintenance doses dex | 3 | 281 | 277 | -0.06 | -0.23 to 0.11 | 0.5 | 0.5 | 0 |
| - No high maintenance doses dex | 3 | 221 | 223 | -0.04 | -0.23 to 0.15 | 0.7 | 0.4 | 0.5 |
| - Loading dose dex | 5 | 251 | 253 | -0.02 | -0.19 to 0.16 | 0.9 | 0.6 | 0 |
| - No loading dose dex | 1 | 251 | 247 | - | - | - | - | - |
| - High and loading doses dex | 1 | 251 | 247 | - | - | - | - | - |
| - Blind | 1 | 251 | 247 | - | - | - | - | - |
| - CABG | 2 | 191 | 193 | 0.01 | -0.19 to 0.21 | 0.9 | 0.6 | 0 |
| - Low risk of bias studies | 1 | 251 | 247 | - | - | - | - | - |

ICU: intensive care unit; Dex: dexmedetomidine; SMD: standardized mean difference; CI: confidence interval; ICU: intensive care unit**;** P: p-value; CABG: coronary artery bypass grafting
