# Supplementary material for: Dexmedetomidine as a Sedative Agent in Critically Ill Patients: A Meta-Analysis of Randomized Controlled Trials
Source: PLoS One. 2013 Dec 31;8(12):e82913. doi: 10.1371/journal.pone.0082913 (PMC3877008; doi:10.1371/journal.pone.0082913)
Supplement: Table S3 — Subanalysis with midazolam as comparator drug (DOCX) [file pone.0082913.s008.docx]

| **Outcome** Comparator MIDAZOLAM | **Number of included trials** | **Dex**  **patients** | **Control**  **patients** | **SMD** | **95% CI** | **P for effect** | **P for heterogeneity** | **I^2^ (%)** |
| --- | --- | --- | --- | --- | --- | --- | --- | --- |
| **ICU stay** |  |  |  |  |  |  |  |  |
| **Overall trials** | **4 trials** | **533** | **413** | **-0.69** | **-1.59 to 0.20** | **0.13** | **<0.001** | **97** |
| - Long term sedation | 2 | 493 | 373 | -0.79 | -2.19 to 0.69 | 0.3 | <0.001 | 99 |
| - Short term sedation | 2 | 40 | 40 | -0.59 | -1.40 to 0.23 | 0.2 | 0.07 | 69 |
| - Daily interruption sedation | 2 | 493 | 373 | -0.79 | -2.19 to 0.69 | 0.3 | <0.001 | 99 |
| - High maintenance doses dex | 2 | 493 | 373 | -0.79 | -2.19 to 0.69 | 0.3 | <0.001 | 99 |
| - No high maintenance doses dex | 2 | 40 | 40 | -0.59 | -1.40 to 0.23 | 0.2 | 0.07 | 69 |
| - Loading dose dex | 2 | 40 | 40 | -0.59 | -1.40 to 0.23 | 0.2 | 0.07 | 69 |
| - No loading dose dex | 2 | 493 | 373 | -0.79 | -2.19 to 0.69 | 0.3 | <0.001 | 99 |
| - High and loading doses dex | 0 | - | - | - | - | - | - | - |
| - Blind | 2 | 493 | 373 | -0.79 | -2.19 to 0.69 | 0.3 | <0.001 | 99 |
| - CABG | 1 | 20 | 20 | - | - | - | - | - |
| - Low risk of bias studies | 2 | 493 | 373 | -0.79 | -2.19 to 0.69 | 0.3 | <0.001 | 99 |
|  |  |  |  |  |  |  |  |  |
| **Time to extubation** |  |  |  |  |  |  |  |  |
| **Overall trials** | **7 trials** | **685** | **564** | **-0.72** | **-1.50 to 0.06** | **0.07** | **<0.001** | **97** |
| - Long term sedation | 3 | 528 | 411 | -0.89 | -2.49 to 0.73 | 0.3 | <0.001 | 99 |
| - Short term sedation | 4 | 157 | 153 | -0.59 | -0.95 to -0.23 | 0.001 | 0.2 | 43 |
| - Daily interruption sedation | 2 | 493 | 373 | -1.29 | -3.54 to 0.95 | 0.3 | <0.001 | 99 |
| - High maintenance doses dex | 2 | 493 | 373 | -1.29 | -3.54 to 0.95 | 0.3 | <0.001 | 99 |
| - No high maintenance doses dex | 4 | 90 | 93 | -0.38 | -0.86 to 0.09 | 0.1 | 0.06 | 59 |
| - Loading dose dex | 3 | 75 | 78 | -0.26 | -0.77 to 0.26 | 0.3 | 0.09 | 59 |
| - No loading dose dex | 3 | 508 | 388 | -1.16 | -2.88 to 0.57 | 0.2 | <0.001 | 99 |
| - High and loading doses dex | 0 | - | - | - | - | - | - | - |
| - Blind | 2 | 493 | 373 | -1.29 | -3.54 to 0.95 | 0.3 | <0.001 | 99 |
| - CABG | 2 | 35 | 35 | -0.86 | -1.35 to -0.36 | 0.001 | 0.9 | 0 |
| - Low risk of bias studies | 2 | 493 | 373 | -1.29 | -3.54 to 0.95 | 0.3 | <0.001 | 99 |

ICU: intensive care unit; Dex: dexmedetomidine; SMD: standardized mean difference; CI: confidence interval; ICU: intensive care unit**;** P: p-value; CABG: coronary artery bypass grafting
