# Supplementary material for: Dexmedetomidine as a Sedative Agent in Critically Ill Patients: A Meta-Analysis of Randomized Controlled Trials
Source: PLoS One. 2013 Dec 31;8(12):e82913. doi: 10.1371/journal.pone.0082913 (PMC3877008; doi:10.1371/journal.pone.0082913)
Supplement: Table S4 — Subanalysis with morphine as comparator drug (DOCX) [file pone.0082913.s009.docx]

| **Outcome** Comparator MORPHINE | **Number of included trials** | **Dex**  **patients** | **Control**  **patients** | **SMD** | **95% CI** | **P for effect** | **P for heterogeneity** | **I^2^ (%)** |
| --- | --- | --- | --- | --- | --- | --- | --- | --- |
| **ICU stay** |  |  |  |  |  |  |  |  |
| Overall trials | 1 trials | 154 | 152 | - | - | - | - | - |
| - Long term sedation | 0 | - | - | - | - | - | - | - |
| - Short term sedation | 1 | 154 | 152 | - | - | - | - | - |
| - Daily interruption sedation | 0 | - | - | - | - | - | - | - |
| - High maintenance doses dex | 0 | - | - | - | - | - | - | - |
| - No high maintenance doses dex | 1 | 154 | 152 | - | - | - | - | - |
| - Loading dose dex | 0 | - | - | - | - | - | - | - |
| - No loading dose dex | 1 | 154 | 152 | - | - | - | - | - |
| - High and loading doses dex | 0 | - | - | - | - | - | - | - |
| - Blind | 0 | - | - | - | - | - | - | - |
| - CABG | 0 | - | - | - | - | - | - | - |
| - Low risk of bias studies | 1 | 154 | 152 | - | - | - | - | - |
|  |  |  |  |  |  |  |  |  |
| **Time to extubation** |  |  |  |  |  |  |  |  |
| Overall trials | 2 trials | 168 | 166 | -0.27 | -0.48 to -0.05 | 0.02 | 0.9 | 0 |
| - Long term sedation | 0 | - | - | - | - | - | - | - |
| - Short term sedation | 2 | 168 | 166 | -0.27 | -0.48 to -0.05 | 0.02 | 0.9 | 0 |
| - Daily interruption sedation | 0 | - | - | - | - | - | - | - |
| - High maintenance doses dex | 0 | - | - | - | - | - | - | - |
| - No high maintenance doses dex | 2 | 168 | 166 | -0.27 | -0.48 to -0.05 | 0.02 | 0.9 | 0 |
| - Loading dose dex | 0 | - | - | - | - | - | - | - |
| - No loading dose dex | 2 | 168 | 166 | -0.27 | -0.48 to -0.05 | 0.02 | 0.9 | 0 |
| - High and loading doses dex | 0 | - | - | - | - | - | - | - |
| - Blind | 1 | 154 | 152 | - | - | - | - | - |
| - CABG | 0 | - | - | - | - | - | - | - |
| - Low risk of bias studies | 1 | 154 | 152 | - | - | - | - | - |
