# Supplementary material for: Dexmedetomidine as a Sedative Agent in Critically Ill Patients: A Meta-Analysis of Randomized Controlled Trials
Source: PLoS One. 2013 Dec 31;8(12):e82913. doi: 10.1371/journal.pone.0082913 (PMC3877008; doi:10.1371/journal.pone.0082913)
Supplement: Table S5 — Subanalysis with placebo as comparator drug (DOCX) [file pone.0082913.s010.docx]

| **Outcome** Comparator PLACEBO | **Number of included trials** | **Dex**  **patients** | **Control**  **patients** | **SMD** | **95% CI** | **P for effect** | **P for heterogeneity** | **I^2^ (%)** |
| --- | --- | --- | --- | --- | --- | --- | --- | --- |
| **ICU stay** |  |  |  |  |  |  |  |  |
| **Overall trials** | **2 trials** | **40** | **40** | **-1.48** | **-4.40 to 1.44** | **0.3** | **<0.001** | **97** |
| - Long term sedation | 0 | - | - | - | - | - | - | - |
| - Short term sedation | 2 | 40 | 40 | -1.48 | -4.40 to 1.44 | 0.3 | <0.001 | 97 |
| - Daily interruption sedation | 0 | - | - | - | - | - | - | - |
| - High maintenance doses dex | 0 | - | - | - | - | - | - | - |
| - No high maintenance doses dex | 2 | 40 | 40 | -1.48 | -4.40 to 1.44 | 0.3 | <0.001 | 97 |
| - Loading dose dex | 2 | 40 | 40 | -1.48 | -4.40 to 1.44 | 0.3 | <0.001 | 97 |
| - No loading dose dex | 0 | - | - | - | - | - | - | - |
| - High and loading doses dex | 0 | - | - | - | - | - | - | - |
| - Blind | 2 | 40 | 40 | -1.48 | -4.40 to 1.44 | 0.3 | <0.001 | 97 |
| - CABG | 2 | 40 | 40 | -1.48 | -4.40 to 1.44 | 0.3 | <0.001 | 97 |
| - Low risk of bias studies | 2 | 40 | 40 | -1.48 | -4.40 to 1.44 | 0.3 | <0.001 | 97 |
|  |  |  |  |  |  |  |  |  |
| **Time to extubation** |  |  |  |  |  |  |  |  |
| **Overall trials** | **5 trials** | **302** | **296** | **-0.50** | **-1.23 to 0.22** | **0.2** | **<0.001** | **91** |
| - Long term sedation | 0 | - | - | - | - | - | - | - |
| - Short term sedation | 5 | 302 | 296 | -0.50 | -1.23 to 0.22 | 0.2 | <0.001 | 91 |
| - Daily interruption sedation | 0 | - | - | - | - | - | - | - |
| - High maintenance doses dex | 1 | 44 | 43 | - | - | - | - | - |
| - No high maintenance doses dex | 4 | 258 | 253 | -0.57 | -1.65 to 0.50 | 0.3 | <0.001 | 93 |
| - Loading dose dex | 5 | 302 | 296 | -0.50 | -1.23 to 0.22 | 0.2 | <0.001 | 91 |
| - No loading dose dex | 0 | - | - | - | - | - | - | - |
| - High and loading doses dex | 1 | 44 | 43 | - | - | - | - | - |
| - Blind | 5 | 302 | 296 | -0.50 | -1.23 to 0.22 | 0.2 | <0.001 | 91 |
| - CABG | 3 | 84 | 83 | -0.95 | -2.44 to 0.55 | 0.2 | <0.001 | 94 |
| - Low risk of bias studies | 3 | 84 | 83 | -0.95 | -2.44 to 0.55 | 0.2 | <0.001 | 94 |

ICU: intensive care unit; Dex: dexmedetomidine; SMD: standardized mean difference; CI: confidence interval; ICU: intensive care unit**;** P: p-value; CABG: coronary artery bypass grafting
