# Supplementary material for: Dexmedetomidine as a Sedative Agent in Critically Ill Patients: A Meta-Analysis of Randomized Controlled Trials
Source: PLoS One. 2013 Dec 31;8(12):e82913. doi: 10.1371/journal.pone.0082913 (PMC3877008; doi:10.1371/journal.pone.0082913)
Supplement: Text S1 — Full PubMed search strategy (DOCX) [file pone.0082913.s011.docx]

**PUBMED SEARCH STRATEGY**

dexmedetomidine AND (randomized controlled trial [pt] OR controlled clinical trial[pt] OR randomized controlled trials[mh] OR random allocation[mh] OR double-blind method[mh] OR single-blind method[mh] OR clinical trial[pt] OR clinical trials[mh] OR (clinical trial[tw] OR ((singl*[tw] OR doubl*[tw] OR trebl*[tw] OR tripl*[tw]) AND (mask*[tw] OR blind[tw])) OR (latin square[tw]) OR placebos[mh] OR placebo*[tw] OR random*[tw] OR research design[mh:noexp] OR follow-up studies[mh] OR prospective studies[mh] OR cross-over studies[mh] OR control*[tw] OR prospectiv*[tw] OR volunteer*[tw]) NOT (animal[mh] NOT human[mh]) NOT (comment[pt] OR editorial[pt] OR meta-analysis[pt] OR practice-guideline[pt] OR review[pt]))
