# Supplementary material for: Dexmedetomidine as a Sedative Agent in Critically Ill Patients: A Meta-Analysis of Randomized Controlled Trials
Source: PLoS One. 2013 Dec 31;8(12):e82913. doi: 10.1371/journal.pone.0082913 (PMC3877008; doi:10.1371/journal.pone.0082913)
Supplement: Text S2 — Major exclusions (DOCX) [file pone.0082913.s012.docx]

**MAJOR EXCLUSIONS**

Database searches, snowballing, and contacts with experts yielded a total of 573 articles (figure 1). Excluding 498 non-pertinent titles or abstracts, we retrieved in complete form and assessed 75 studies according to the selection criteria.

Forty-eight studies were further excluded because of our prespecified exclusion criteria. Two studies were excluded because there were no outcome data and further details could not be obtained by the authors [supplemental references 1,2], two studies because dexmedetomidine was not intravenously administered [supplemental references 3-4], eight studies because dexmedetomidine was administered as a sedative agent in non-ICU, non surgical settings [supplemental references 5-12], one study because dexmedetomidine was administered on healthy volunteers [supplemental references 13], two studies because the data were included in a larger or previous publications [supplemental references 14,15] , four because patients were not mechanical ventilated during the ICU stay [supplemental references 16-19], two studies because non adult studies [supplemental references 20,21], one study because all groups received dexmedetomidine and there was no control group [supplemental reference 22] and 26 studies because dexmedetomidine was administered only intraoperatively or postoperatively in non-ICU settings [supplemental references 23-48],
