# Supplementary material for: Dexmedetomidine as a Sedative Agent in Critically Ill Patients: A Meta-Analysis of Randomized Controlled Trials
Source: PLoS One. 2013 Dec 31;8(12):e82913. doi: 10.1371/journal.pone.0082913 (PMC3877008; doi:10.1371/journal.pone.0082913)
Supplement: Text S3 — References of the excluded studies (DOCX) [file pone.0082913.s013.docx]

**REFERENCES OF THE EXCLUDED STUDIES**

[1] Talke P, Li J, Jain U, Leung J, Drasner K, et al. (1995) [Effects of perioperative dexmedetomidine infusion in patients undergoing vascular surgery. The Study of Perioperative Ischemia Research Group.](http://www.ncbi.nlm.nih.gov/pubmed/7879930) Anesthesiology 82: 620-33.

[2] Kaneko T. (2008) [Postoperative management of carotid endarterectomy with dexmedetomidine--a comparison with propofol.](http://www.ncbi.nlm.nih.gov/pubmed/18546896) Masui 57: 696-703.

[3] Esmaoglu A, Yegenoglu F, Akin A, Turk CY. (2010) [Dexmedetomidine added to levobupivacaine prolongs axillary brachial plexus block.](http://www.ncbi.nlm.nih.gov/pubmed/20889939) Anesth Analg 111: 1548-51.

[4] Bajwa SJ, Bajwa SK, Kaur J, Singh G, Arora V, et al. (2011) Dexmedetomidine and clonidine in epidural anaesthesia: A comparative evaluation. Indian J Anaesth 55: 116-121.

[5] Zor F, Ozturk S, Bilgin F, Isik S, Cosar A. (2010) Pain relief during dressing changes of major adult burns: ideal analgesic combination with ketamine. [Burns](http://www.ncbi.nlm.nih.gov/pubmed?term=zor%20dexmedetomidine) 36: 501-5.

[6] Takimoto K, Ueda T, Shimamoto F, Kojima Y, Fujinaga Y, et al. (2011) Sedation with dexmedetomidine hydrochloride during endoscopic submucosal dissection of gastric cancer. [Dig Endosc](http://www.ncbi.nlm.nih.gov/pubmed?term=takimoto%20dexmedetomidine) 23: 176-81.

[7] Gündüz M, Sakalli S, Güneş Y, Kesiktaş E, Ozcengiz D, et al. (2011) Comparison of effects of ketamine, ketamine-dexmedetomidine and ketamine-midazolam on dressing changes of burn patients. J Anaesthesiol Clin Pharmacol 27: 220-224.

[8] Vázquez-Reta JA, Jiménez Ferrer MC, Colunga-Sánchez A, Pizarro-Chávez S, Vázquez-Guerrero AL, et al. (2011) Midazolam versus dexmedetomidine for sedation for upper gastrointestinal endoscopy. Rev Gastroenterol Mex 76: 13-18.

[9] Muller S, Borowics SM, Fortis EA, Stefani LC, Soares G, et al. (2008) Clinical efficacy of dexmedetomidine alone is less than propofol for conscious sedation during ERCP. [Gastrointest Endosc](http://www.ncbi.nlm.nih.gov/pubmed?term=muller%20dexmedetomidine) 67: 651-9.

[10] Dere K, Sucullu I, Budak ET, Yeyen S, Filiz AI, et al. (2010) A comparison of dexmedetomidine versus midazolam for sedation, pain and hemodynamic control, during colonoscopy under conscious sedation. [Eur J Anaesthesiol](http://www.ncbi.nlm.nih.gov/pubmed?term=dere%20dexmedetomidine) 27: 648-52.

[11] Bergese SD, Patrick Bender S, McSweeney TD, Fernandez S, Dzwonczyk R, et al. (2010) [A comparative study of dexmedetomidine with midazolam and midazolam alone for sedation during elective awake fiberoptic intubation.](http://www.ncbi.nlm.nih.gov/pubmed/20206849) J Clin Anesth 22: 35-40.

[12] Turan A, Sen H, Sizlan A, Yanarateş O, Ozkan S, et al. (2011) [Dexmedetomidine: an alternative for epidural anesthesia in tension-free vaginal-tape surgery.](http://www.ncbi.nlm.nih.gov/pubmed/21416311) J Anesth 25: 386-91.

[13] Kasuya Y, Govinda R, Rauch S, Mascha EJ, Sessler DI, et al. (2009) The correlation between bispectral index and observational sedation scale in volunteers sedated with dexmedetomidine and propofol. [Anesth Analg](http://www.ncbi.nlm.nih.gov/pubmed/19923507) 109: 1811-5.

[14] Venn RM, Bradshaw CJ, Spencer R, Brealey D, Caudwell E, et al. (1999) [Preliminary UK experience of dexmedetomidine, a novel agent for postoperative sedation in the intensive care unit.](http://www.ncbi.nlm.nih.gov/pubmed/10594409) Anaesthesia 54: 1136-42.

[15] Dasta JF, Kane-Gill SL, Pencina M, Shehabi Y, Bokesch PM, et al. (2010) A cost-minimization analysis of dexmedetomidine compared with midazolam for long-term sedation in the intensive care unit. [Crit Care Med](http://www.ncbi.nlm.nih.gov/pubmed?term=dasta%20dexmedetomidine) 38: 497-503.

[16] Akin S, Aribogan A, Arslan G. (2008) Dexmedetomidine as an adjunct to epidural analgesia after abdominal surgery in elderly intensive care patients: a prospective, double-blind, clinical trial. Curr Ther Res Clin Exp 69: 16-28.

[17] Talke P, Chen R, Thomas B, Aggarwall A, Gottlieb A, et al. (2000) The hemodynamic and adrenergic effects of perioperative dexmedetomidine infusion after vascular surgery. Anesth Analg 90: 834-9.

[18] Wahlander S, Frumento RJ, Wagener G, Saldana-Ferretti B, Joshi RR, et al. (2005) A prospective, double-blind, randomized, placebo-controlled study of dexmedetomidine as an adjunct to epidural analgesia after thoracic surgery. J Cardiothorac Vasc Anesth 19: 630-5.

[19] Huang Z, Chen YS, Yang ZL, Liu JY. (2012) Dexmedetomidine versus midazolam for the sedation of patients with non-invasive ventilation failure. Intern Med 51: 2299-305.

[20] Burbano NH, Otero AV, Berry DE, Orr RA, Munoz RA. (2012) Discontinuation of prolonged infusions of dexmedetomidine in critically ill children with heart disease. Intensive Care Med 38: 300-7.

[21] Le KN, Moffett BS, Ocampo EC, Zaki J, Mossad EB. (2011) Impact of dexmedetomidine on early extubation in pediatric cardiac surgical patients. Intensive Care Med 37: 686-90.

[22] Mahmoud M, Sadhasivam S, Salisbury S, Nick TG, Schnell B, et al. (2010) [Susceptibility of transcranial electric motor-evoked potentials to varying targeted blood levels of dexmedetomidine during spine surgery.](http://www.ncbi.nlm.nih.gov/pubmed/20460997) Anesthesiology 112: 1364-73.

[23] Basar H, Akpinar S, Doganci N, Buyukkocak U, Kaymak C, et al. (2008) [The effects of preanesthetic, single-dose dexmedetomidine on induction, hemodynamic, and cardiovascular parameters.](http://www.ncbi.nlm.nih.gov/pubmed/18929283) J Clin Anesth 20: 431-6.

[24] Elcicek K, Tekin M, Kati I. (2010) [The effects of intravenous dexmedetomidine on spinal hyperbaric ropivacaine anesthesia.](http://www.ncbi.nlm.nih.gov/pubmed/20467879) J Anesth 24: 544-8.

[25] Bayram A, Esmaoglu A, Akin A, Baskol G, Aksu R, et al. (2011) [The effects of intraoperative infusion of dexmedetomidine on early renal function after percutaneous nephrolithotomy.](http://www.ncbi.nlm.nih.gov/pubmed/21827441) Acta Anaesthesiol Scand 55: 539-44.

[26] Chu KS, Wang FY, Hsu HT, Lu IC, Wang HM, et al. (2010) [The effectiveness of dexmedetomidine infusion for sedating oral cancer patients undergoing awake fibreoptic nasal intubation.](http://www.ncbi.nlm.nih.gov/pubmed/19550337) Eur J Anaesthesiol 27: 36-40.

[27] [Sitilci AT, Ozyuvacı E, Alkan Z, Demirgan S, Yiğit O. (2010) The effect of perioperative infused dexmedetomidine on postoperative analgesic consumption in mastoidectomy operations.](http://www.ncbi.nlm.nih.gov/pubmed/20865582) Agri 22: 109-16.

[28] [Rutkowska K](http://www.ncbi.nlm.nih.gov/pubmed?term=Rutkowska%20K%5BAuthor%5D&cauthor=true&cauthor_uid=19550340), [Knapik P](http://www.ncbi.nlm.nih.gov/pubmed?term=Knapik%20P%5BAuthor%5D&cauthor=true&cauthor_uid=19550340), [Misiolek H](http://www.ncbi.nlm.nih.gov/pubmed?term=Misiolek%20H%5BAuthor%5D&cauthor=true&cauthor_uid=19550340). (2009) The effect of dexmedetomidine sedation on brachial plexus block in patients with end-stage renal disease. [Eur J Anaesthesiol](http://www.ncbi.nlm.nih.gov/pubmed?term=rutkowska%20dexmedetomidine) 26: 851-5.

[29] Bekker A, Sturaitis M, Bloom M, Moric M, Golfinos J, et al. (2008) [The effect of dexmedetomidine on perioperative hemodynamics in patients undergoing craniotomy.](http://www.ncbi.nlm.nih.gov/pubmed/18806050) Anesth Analg 107: 1340-7.

[30] Turgut N, Turkmen A, Ali A, Altan A. (2009) [Remifentanil-propofol vs dexmedetomidine-propofol--anesthesia for supratentorial craniotomy.](http://www.ncbi.nlm.nih.gov/pubmed/19266828) Middle East J Anesthesiol 20: 63-70.

[31] Mizrak A, Koruk S, Bilgi M, Kocamer B, Erkutlu I, et al. (2010) [Pretreatment with dexmedetomidine or thiopental decreases myoclonus after etomidate: a randomized, double-blind controlled trial.](http://www.ncbi.nlm.nih.gov/pubmed/20018300) J Surg Res 159: 11-6.

[32] Kida K, Ohtani N, Shoji K, Yasui Y, Masaki E. (2008) [Postoperative pain status after intraoperative systemic dexmedetomidine and epidural neostigmine in patients undergoing lower abdominal surgery.](http://www.ncbi.nlm.nih.gov/pubmed/18838046) Eur J Anaesthesiol 25: 869-75.

[33] Candiotti KA, Bergese SD, Bokesch PM, Feldman MA, Wisemandle W, et al. (2010) [Monitored anesthesia care with dexmedetomidine: a prospective, randomized, double-blind, multicenter trial.](http://www.ncbi.nlm.nih.gov/pubmed/19713256) Anesth Analg 110: 47-56.

[34] Kaya FN, Yavascaoglu B, Turker G, Yildirim A, Gurbet A, et al. (2010) [Intravenous dexmedetomidine, but not midazolam, prolongs bupivacaine spinal anesthesia.](http://www.ncbi.nlm.nih.gov/pubmed/20039221) Can J Anaesth 57: 39-45.

[35] Turan A, Wo J, Kasuya Y, Govinda R, Akça O, et al. (2010) [Effects of dexmedetomidine and propofol on lower esophageal sphincter and gastroesophageal pressure gradient in healthy volunteers.](http://www.ncbi.nlm.nih.gov/pubmed/20032699) Anesthesiology 112: 19-24.

[36] Ayoglu H, Yapakci O, Ugur MB, Uzun L, Altunkaya H, et al. (2008) [Effectiveness of dexmedetomidine in reducing bleeding during septoplasty and tympanoplasty operations.](http://www.ncbi.nlm.nih.gov/pubmed/18929284) J Clin Anesth 20: 437-41.

[37] Al-Metwalli RR, Mowafi HA, Ismail SA, Siddiqui AK, Al-Ghamdi AM, et al. (2008) Effect of intra-articular dexmedetomidine on postoperative analgesia after arthroscopic knee surgery. [Br J Anaesth](http://www.ncbi.nlm.nih.gov/pubmed?term=al-metwalli%20dexmedetomidine) 101: 395-9.

[38] Lin TF, Yeh YC, Lin FS, Wang YP, Lin CJ, et al. (2009) [Effect of combining dexmedetomidine and morphine for intravenous patient-controlled analgesia.](http://www.ncbi.nlm.nih.gov/pubmed/18987053) Br J Anaesth 102: 117-22.

[39] Ghali A, Mahfouz AK, Ihanamäki T, El Btarny AM. (2011) [Dexmedetomidine versus propofol for sedation in patients undergoing vitreoretinal surgery under sub-Tenon's anesthesia.](http://www.ncbi.nlm.nih.gov/pubmed/21655014) Saudi J Anaesth 5: 36-41.

[40] Kunisawa T, Nagata O, Nagashima M, Mitamura S, Ueno M, et al. (2009) [Dexmedetomidine suppresses the decrease in blood pressure during anesthetic induction and blunts the cardiovascular response to tracheal intubation.](http://www.ncbi.nlm.nih.gov/pubmed/19464613) J Clin Anesth 21: 194-9.

[41] Yagmurdur H, Ozcan N, Dokumaci F, Kilinc K, Yilmaz F, et al. (2008) [Dexmedetomidine reduces the ischemia-reperfusion injury markers during upper extremity surgery with tourniquet.](http://www.ncbi.nlm.nih.gov/pubmed/18656769) J Hand Surg Am 33: 941-7.

[42] Tufanogullari B, White PF, Peixoto MP, Kianpour D, Lacour T, et al. (2008) Dexmedetomidine infusion during laparoscopic bariatric surgery: the effect on recovery outcome variables. [Anesth Analg](http://www.ncbi.nlm.nih.gov/pubmed?term=tufanogullari%20dexmedetomidine) 106: 1741-8.

[43] Zeyneloglu P, Pirat A, Candan S, Kuyumcu S, Tekin I, et al. Dexmedetomidine causes prolonged recovery when compared with midazolam/fentanyl combination in outpatient shock wave lithotripsy. Eur J Anaesthesiol 25: 961-7.

[44] Uyar AS, Yagmurdur H, Fidan Y, Topkaya C, Basar H. (2008) Dexmedetomidine attenuates the hemodynamic and neuroendocrinal responses to skull-pin head-holder application during craniotomy. J Neurosurg Anesthesiol 20: 174-9.

[45] Dogan R, Erbek S, Gonencer HH, Erbek HS, Isbilen C, et al. (2010) Comparison of local anaesthesia with dexmedetomidine sedation and general anaesthesia during septoplasty. Eur J Anaesthesiol 27: 960-964.

[46] Uzümcügil F, Canbay O, Celebi N, Karagoz AH, Ozgen S. (2008) Comparison of dexmedetomidine-propofol vs. fentanyl-propofol for laryngeal mask insertion. Eur J Anaesthesiol 25: 675-80.

[47] Richa F, Yazigi A, Sleilaty G, Yazbeck P. (2008) Comparison between dexmedetomidine and remifentanil for controlled hypotension during tympanoplasty. Eur J Anaesthesiol 25: 369-74.

[48] Sidorowicz M, Owczuk R, Kwiecińska B, Wujtewicz MA, Wojciechowski J, et al. (2009) Dexmedetomidine sedation for carotid endarterectomy. Anestezjol Intens Ter 41: 78-83.
